# Comparative Mapping Between Coho Salmon (*Oncorhynchus kisutch*) and Three Other Salmonids Suggests a Role for Chromosomal Rearrangements in the Retention of Duplicated Regions Following a Whole Genome Duplication Event

**DOI:** 10.1534/g3.114.012294

**Published:** 2014-07-21

**Authors:** Miyako Kodama, Marine S. O. Brieuc, Robert H. Devlin, Jeffrey J. Hard, Kerry A. Naish

**Affiliations:** * School of Aquatic and Fishery Sciences, University of Washington, Seattle, Washington 98105; † Fisheries and Oceans Canada, West Vancouver, British Columbia, V7K 1N6, Canada; ‡ National Marine Fisheries Service, Northwest Fisheries Science Center, Seattle, Washington 98112

**Keywords:** chromsome rearrangements, comparative genome mapping, RAD sequencing, salmon, whole genome duplication

## Abstract

Whole genome duplication has been implicated in evolutionary innovation and rapid diversification. In salmonid fishes, however, whole genome duplication significantly pre-dates major transitions across the family, and re-diploidization has been a gradual process between genomes that have remained essentially collinear. Nevertheless, pairs of duplicated chromosome arms have diverged at different rates from each other, suggesting that the retention of duplicated regions through occasional pairing between homeologous chromosomes may have played an evolutionary role across species pairs. Extensive chromosomal arm rearrangements have been a key mechanism involved in re-dipliodization of the salmonid genome; therefore, we investigated their influence on degree of differentiation between homeologs across salmon species. We derived a linkage map for coho salmon and performed comparative mapping across syntenic arms within the genus *Oncorhynchus*, and with the genus *Salmo*, to determine the phylogenetic relationship between chromosome arrangements and the retention of undifferentiated duplicated regions. A 6596.7 cM female coho salmon map, comprising 30 linkage groups with 7415 and 1266 nonduplicated and duplicated loci, respectively, revealed uneven distribution of duplicated loci along and between chromosome arms. These duplicated regions were conserved across syntenic arms across *Oncorhynchus* species and were identified in metacentric chromosomes likely formed ancestrally to the divergence of *Oncorhynchus* from *Salmo*. These findings support previous studies in which observed pairings involved at least one metacentric chromosome. Re-diploidization in salmon may have been prevented or retarded by the formation of metacentric chromosomes after the whole genome duplication event and may explain lineage-specific innovations in salmon species if functional genes are found in these regions.

Whole genome duplication (WGD) is a mutational mechanism that can serve as a primary driver of evolutionary novelty (Ohno 1970; Zhang 2003; Crow and Wagner 2006; Lynch 2007; Edger and Pires 2009). Changes in ploidy levels after WGD can lead to dramatic alterations at the cellular and phenotypic level (Mayfield-Jones *et al.* 2013) and provide additional genetic variation for mutation, drift, and selection to act on. These evolutionary processes can result in new adaptations and species diversification (Van De Peer *et al.* 2009; Storz *et al.* 2013). Genome sequencing projects are increasingly revealing that WGD is widespread in many key lineages, such as flowering plants and vertebrates, and represents an ongoing phenomenon in many species (Otto and Whitton 2000; Van De Peer *et al.* 2009). Understanding the processes governing the return to a diploid mode—diploidization—by comparing the genomes of species descended from a WGD event can provide insights into the event’s role in evolutionary innovation and persistence of duplicated regions (Jaillon *et al.* 2009; Mayfield-Jones *et al.* 2013).

The stabilization of the duplicated genome through diploidization can be achieved by rearrangements (such as translocations, fissions, fusions, and transpositions), gene loss, and sequence deletion and divergence (Hufton and Panopoulou 2009; Schubert and Lysak 2011). These processes tend to reduce the similarity of the duplicated ohnologs (Wolfe 2001), and the homeologous chromosomes resulting from WGD, but the exact mechanisms vary across lineages (Hufton and Panopoulou 2009). Whole genome duplication has been frequently implicated in evolutionary innovation in eukaryotic genomes of paleopolyploids (ancient polyploids) (Ohno 1999; Lynch and Conery 2000; Jaillon *et al.* 2004; Cañestro *et al.* 2013), but evidence in plants suggests that the rate of diversification and extinction of neopolyploids can be lower than that of related diploid lineages (Mayrose *et al.* 2011). Increasing the number of studies on mesopolyploids—organisms in the intermediate process of diploidization (Mayfield-Jones *et al.* 2013)—will provide a clearer understanding of contribution of WGD events to evolutionary innovation.

Salmonid fishes are descended from a whole genome duplication event in an autotetraploid ancestor (Allendorf and Thorgaard 1984), distinct from the second round of duplication (2R) that occurred basal to the vertebrate tree and the third round (3R) early in the evolution of the teleosts 225 to 333 million years ago (Hurley *et al.* 2007; Postlethwait 2007; Santini *et al.* 2009; Near *et al.* 2012). This fourth round (4R) of duplication was recently estimated as occurring 88–103 million years ago (Macqueen and Johnston 2014; see also Crête-Lafrenière *et al.* 2012; Alexandrou *et al.* 2013; Berthelot *et al.* 2014). Although the genomes of these species are returning to a stable diploid state through chromosomal rearrangements and divergence of homeologous chromosomes, evidence of tetrasomic inheritance in males and extensive rearrangements among chromosomes has shown that restoration of diploidy is not yet complete (Wright *et al.* 1983; Allendorf and Thorgaard 1984; Allendorf and Danzmann 1997). Comparative genome sequencing between ohnologs in rainbow trout has revealed extensive collinearity between the duplicated chromosomes, characterized by loss of approximately half the protein-coding regions through pseudogenization but retention of most of the duplicated miRNA genes (Berthelot *et al.* 2014).

The role of the WGD event in salmonid trait innovation and diversification is unclear. Recent evidence based on molecular clock estimates suggest that duplication is unlinked to a major transition in life history, anadromy (Alexandrou *et al.* 2013; Macqueen and Johnston 2014), and preceded rapid species diversification by several million years (Berthelot *et al.* 2014; Macqueen and Johnston 2014). Rather, both transitions appear to correspond with climate cooling (Macqueen and Johnston 2014). Re-diploidization has been subsequently characterized as a gradual process unlinked to significant genome rearrangements (Berthelot *et al.* 2014). However, it has also been argued that the duplication event might have provided the raw material for evolution to act on, and that differential divergence of duplicated regions might have promoted speciation at varying time points (Macqueen and Johnston 2014). Large-scale genome characterization in the salmonids is increasingly revealing the location of genes or regions that may have played a role in adaptation and diversification (Davidson *et al.* 2010; Bourret *et al.* 2013; Larson *et al.* 2013). Therefore, it is important to combine these studies with an understanding of the mechanism and timing of divergence between homeologous chromosome arms across salmon lineages so that it will be possible to understand how duplication played a role in evolution of salmon, and whether key genomic regions might explain innovation across a subset of species.

It has been known for some time that one of the key mechanisms for diploidization within the subfamilies Coregoninae and Salmoninae (which includes *Salmo*, *Salvelinu*s, and *Oncorhynchus*) has occurred through Robertsonian rearrangements of whole chromosome arms (Ohno 1999; Phillips and Ráb 2001). Most chromosome arms are syntenic between Salmoninae species, and the combined efforts of genome mapping and karyotyping have permitted alignment of chromosome arms among several species within this subfamily (Danzmann *et al.* 2005; Phillips *et al.* 2009; Lubieniecki *et al.* 2010; Lien *et al.* 2011; Timusk *et al.* 2011; Guyomard *et al.* 2012; Naish *et al.* 2013; Phillips *et al.* 2013). Chromosome arm number is largely conserved (NF = approximately 100) but the numbers of chromosomes vary substantially across species as a result of the Robertsonian rearrangements (Phillips and Ráb 2001). The exception is Atlantic salmon, with reduced chromosome arm number compared with the other species (NF = 72–74). However, large syntentic blocks within the arms of this species correspond to whole arms in other members of the Salmoninae, making comparative studies feasible across this subfamily as a whole.

Comparative mapping between Chinook salmon (*O. tshawytscha*) and rainbow trout (*O. mykiss*) has revealed evidence for the retention of at least eight metacentric chromosomes and four acrocentric chromosomes that are ancestral to species divergence within the genus *Oncorhynchus* (Naish *et al.* 2013; Phillips *et al.* 2013). One of the eight metacentric chromosomes and one of the acrocentric chromosomes are also ancestral to the divergence between *Salmo* and *Oncorhynchus*. There is also further evidence for another ancestral metacentric and an ancestral acrocentric chromosome, but these have undergone subsequent rearrangements within at least one descendant species (Naish *et al.* 2013; Ostberg *et al.* 2013; Brieuc *et al.* 2014). High-density linkage maps have revealed that recently diverged or undifferentiated duplicated loci are not uniformly distributed among chromosomes (Atlantic salmon, Lien *et al.* 2011; Chinook salmon, Brieuc *et al.* 2014), and the biased distribution of duplicated loci along chromosome arms provides evidence that pairs of homeologous arms have diverged at different rates from each other (Brieuc *et al.* 2014). This finding confirms observations from previous studies conducted with lower marker densities (for example, Danzmann *et al.* 2005; Guyomard *et al.* 2012). Intriguingly, homeologous pairings have been observed to include at least one metacentric chromosome (Wright *et al.* 1983), and duplicated markers map to such chromosomes (Brieuc *et al.* 2014), supporting the view that metacentric chromosomes play an important role in homeologous pairing (Phillips *et al.* 2009; Brieuc *et al.* 2014). These observations raise the interesting possibility that the evolutionary timing of metacentric chromosome formation during re-diploidization after the WGD event might influence the retention, and hence the evolutionary role, of duplicated regions across species. Therefore, by comparing chromosomal arrangements and distribution of duplicated regions across salmon species, we aim to provide a contextual framework for the further investigation of loci involved in species diversification.

The development of a high-density linkage map for a less-described salmon species will contribute further information regarding chromosome rearrangements that have already been defined in several salmon species and enhance our understanding of the timing of these arrangements in a phylogenetic context. Examining the distribution of duplicated regions across individual chromosome arms in a second species—beyond Chinook salmon (Brieuc *et al.* 2014)—will also facilitate an understanding of the relationship between timing of metacentric chromosome formation and diversification between homeologs. Coho salmon (*Oncorhynchus kisutch*) is a species whose genome has not been extensively described to date. A low-density linkage map of coho salmon has been constructed using microsatellites (McClelland and Naish 2008), but this map is not sufficiently resolved to study the consequences of WGD because there is a low number of duplicated loci mapped. A high-density map in this species is feasible, given recently emerged sequencing technologies (for example, Baird *et al.* 2008). Coho and Chinook salmon are sister species (Crête-Lafrenière *et al.* 2012); therefore, comparative mapping across coho and Chinook salmon, as well as more divergent species in the genus *Oncorhynchus* and *Salmo*, will help validate the hypotheses and provide more robust evidence on the process of chromosomal evolution following WGD.

The aim of our research is to determine the relationship between chromosome arrangements and the retention of recently diverged or undifferentiated duplicated regions by deriving a linkage map for coho salmon and comparing this map with those of Chinook salmon, rainbow trout, and Atlantic salmon. We therefore constructed high-density linkage maps for coho salmon using restriction site–associated DNA (RAD) sequencing (Baird *et al.* 2008). By achieving this objective, we also produced a reference database of RAD markers that can be used for alignment of sequences generated in future work, and described in detail the properties of the coho linkage map. Coho salmon chromosome arms were identified by comparative mapping with Chinook salmon using markers in common between the species, and whole chromosome arm homologies were described across species to improve our current understanding of chromosome arm rearrangement within the genera *Oncorhynchus* and *Salmo*. Linkage groups representing homeologous chromosome arms in coho salmon were discovered using duplicated markers, and regions of duplicated markers were compared across species to determine the extent to which these regions were conserved across lineages. By identifying genomic regions that are in the process of diploidization and linking these regions to chromosomal rearrangements, we aim to provide the basis for determining the role of duplication in maintaining ongoing polymorphisms and explaining processes of diversification across Salmoninae species.

## Materials and Methods

### Justification and description of sample collection and experimental crosses

A two-step approach was used to develop genomic resources and construct linkage maps. First, RAD sequences from individuals sampled from multiple populations were used to construct a reference database for aligning loci across mapping families. This reference database was screened for errors, duplicated loci, and repeat regions following approaches described by Brieuc *et al.* (2014), and loci were subsequently named to ensure consistency across mapping families. Second, specific cross types were used to perform the mapping: gynogenetic haploid crosses were used to map both duplicated and nonduplicated loci, and diploid crosses were used to construct sex-specific maps.

The reference database of RAD loci was constructed using sequences from 583 individuals representing four populations in the Pacific Northwest of the United States and Canada: (1) the Washington Department of Fish and Wildlife’s (WDFW) Wallace River Hatchery (47°87′N, 121°71′W); (2) the Domsea broodstock population, which originated in 1973 and 1974 from Wallace River Hatchery; (3) Bingham Creek (47°15′N, 123°40′W), a tributary to the Satsop River in the Southwest Washington Coast/Lower Columbia ESU; and (4) Chehalis River located in British Columbia, Canada (49°29′N, 121°94′W).

An initial framework map was constructed using two haploid crosses (haploid family 1 and 2) comprising 64 and 62 individuals, respectively. These types of crosses have the advantage of identifying duplicated loci, because these loci will appear as heterozygotes in the offspring if they are polymorphic, while nonduplicated loci will be homozygous. Haploid families were created at the University of Washington hatchery facility (47°65′N, 122°31′W), following the protocol of Thorgaard *et al.* (1983). Embryos were collected before hatching and preserved in 100% ethanol.

Sex-specific maps were created using two F3 outbred diploid crosses and one outbred diploid cross. Specifically, F3 diploid crosses were created from a cultured line originally derived from an outbred cross between two populations in Washington State (McClelland and Naish 2010). F0 males were collected from Bingham Creek in Southwest Washington (47°15′N, 123°40′W). F0 females were obtained from the Domsea broodstock farm. Two F3 crosses were established in December 2010 by mating two F2 full-sibs to create one family, and two F2 half-sibs to create the other. The two families comprised 55 and 67 offspring, respectively, (diploid family 1 and 2). An additional diploid outbred cross was created from an aquaculture population using coho salmon derived from the Chehalis River located in British Columbia, Canada (49°29′N, 121°94′W). Specifically, cultured individuals were repeatedly backcrossed with wild individuals from the Chehalis River for six generations, and diploid crosses were created in January 2011. One diploid family from these crosses comprising 99 individuals was used for further analyses (diploid family 3).

### DNA extraction, sequencing, and amplification of sex-linked markers

Genomic DNA from the sampled individuals was extracted using the DNeasy extraction kit (QIAGEN, Valencia, CA) following the manufacturer’s procedures. The DNA was digested with *Sbf*I, and a 6-nucleotide barcode was added to each sample for individual identification following protocols described by Baird *et al.* (2008). Between 24 and 36 individuals were pooled in a single library and sequenced with 100-bp single-read lengths using the Illumina HiSequation 2000 sequencer. The sequences were separated by individual using PROCESS_RADTAGS implemented in STACKS (Catchen *et al.* 2011, 2013). Because the quality score of sequences decreased beyond 74 nucleotides, sequences were trimmed to 74 nucleotides to remove low-quality sequences. A locus was defined as a 74-nucleotide RAD sequence for the purpose of this study.

Genetic sex was determined in the two diploid families (diploid family 1 and 2) using a Y-linked growth hormone pseudogene (GH5 and GH6) (Devlin *et al.* 2001) and sex-determining gene, sdY (sdY E2S1 and sdY E2AS4) (Yano *et al.* 2012). Polymerase chain reactions were performed for each set of primers using a QIAGEN Multiplex PCR kit. Specifically, reaction mixtures consisted of 10–200 ng genomic DNA, 1× QIAGEN Multiplex PCR Master Mix, 0.25 μM of GH5 and GH6, or 0.4 μM of sdY E2S1 and sdY E2AS4, comprising a total volume of 10 μl. Cycling conditions consisted of a 15-min initial activation step at 95°, 30 cycles of 30-s denaturing step at 94°, 90-s annealing step at 60°, a 60-s extension step at 72°, and a 10-min final extension step at 72°.

### Reference database of RAD loci

RAD loci that are found within repeat regions, and loci containing repeat units, can confound the identification of unique loci. Therefore, a reference sequence database comprising a set of pre-screened RAD loci was first created from the survey of four populations following bioinformatic procedures fully described by Brieuc *et al.* (2014). This database served as a resource for aligning loci across studies. In brief, sequences from all 583 individuals sampled across the four populations described previously were extracted using STACKS 0.9995 (Catchen *et al.* 2011). Both monomorphic and polymorphic loci that were sequenced with a depth greater than 5× in more than 496 individuals (85%) were retained in a temporary database and used for further screening.

Loci in the temporary database that corresponded to repeat regions and loci containing repeat units were removed using two alignment-based strategies following the protocol of Brieuc *et al.* (2014). First, loci in the temporary database were aligned against themselves using BOWTIE (Langmead *et al.* 2009) by allowing a maximum of three nucleotide mismatches per locus. A locus that aligned to several loci, or a locus that did not align to itself, was removed from the temporary database. Then, a BLAST search (Altschul *et al.* 1990) of the temporary database was conducted against itself. Loci that did not return a match, or loci where the best matches were not themselves, were removed from the temporary database.

Using the updated temporary database of RAD loci, polymorphic duplicated loci were identified based on two haploid families. First, sequences from these haploid families were aligned to the temporary database using BOWTIE, allowing a maximum of three nucleotide mismatches per locus. Sequences from the haploid individuals that aligned to more than one locus in the database could not be confidently relied on in further analyses; they were thus identified as “black-listed” loci and removed from the temporary database. Subsequently, polymorphic loci sequenced with a depth greater than 10× per haploid individual were identified using STACKS and retained for further screening. Among these polymorphic loci, a locus was identified as being putatively duplicated when more than one haploid offspring in a family was heterozygous at this particular locus (Brieuc *et al.* 2014). A final reference database comprising named duplicated and nonduplicated loci, as well as loci removed from the alignment-based screening steps and “black-listed” loci, was created.

### Genotyping of individuals in map crosses

Haploid individuals were genotyped at both nonduplicated and duplicated loci. Sequences from all haploid individuals were aligned to the nonduplicated and duplicated loci from the final reference database using BOWTIE by allowing a maximum of three nucleotide mismatches per locus. In haploids, we have shown reliable identification of single loci that have up to three SNPs; we have confirmed this result with genome mapping. To remain consistent, we used up to three mismatches so that we could differentiate between nonduplicated loci and duplicated loci. Both this study and a previous one (Brieuc *et al.* 2014) have shown that very reliable linkage results can be obtained in haploids using these criteria. Polymorphic loci sequenced with a depth greater than 10× per individual were identified using STACKS. Both nonduplicated and duplicated markers in the haploid families were used for mapping, described below and following the protocol of Brieuc *et al.* (2014). Polymorphic duplicated loci were mapped when one of the paralogs was polymorphic (OPP: one paralog polymorphic, parental genotypes aa and ab or aa and bc) or when both paralogs were polymorphic (BPP: parental genotypes ab and ac or ab and cd; see Table 1 in Brieuc *et al.* 2014).

**Table 1:** Description of the Coho salmon consensus linkage map constructed with haploid and diploid female parents and comparison with chromosome arms of Chinook salmon, rainbow trout, and Atlantic salmon

Coho Linkage Group	Size (cM)	Number of Markers	Coho Linkage Arms	Chinook Chromosome (Philips *et al.* 2013; Brieuc *et al.* 2014)	Rainbow Trout Chromosome (Philips *et al.* 2009; Brieuc *et al.* 2014)	Atlantic Salmon Chromosome (Danzmann *et al.* 2008; Philips *et al.* 2009)	Chromosomal Rearrangement Conserved Across Species
Co01	267.51	393	Co01a	Ots02p	Omy17p	Ssa02q	B, metacentric
			Co01b	Ots02q	Omy17q	Ssa12qb^†^	
Co02	248.6	344	Co02a	Ots03p	Omy03p	Ssa02p	B, metacentric
			Co02b	Ots03q	Omy03q	Ssa25	
Co03	274.51	360	Co03a	Ots04p	Omy06p	Ssa24	B, metacentric
			Co03b	Ots04q	Omy06q	Ssa26	
Co04	250.26	395	Co04a	Ots06p	Omy01p	Ssa16qa	B, metacentric
			Co04b	Ots06q	Omy01q	Ssa18qa	
Co05	268.59	376	Co05a	Ots07p	Omy07p	Ssa17qb	B, metacentric
			Co05b	Ots07q	Omy07q	Ssa22	
Co06	267.8	415	Co06a	Ots09p	Omy12p	Ssa13qb	B, metacentric
			Co06b	Ots09q	Omy12q	Ssa03q	
Co07	211.09	241	Co07a	Ots11p*	Omy19p	Ssa04p	B, metacentric
			Co07b	Ots11q	Omy19q	Ssa01p	
Co08	295.21	382	Co08a	Ots12p	Omy11p&q	Ssa20qa^#^	C, metacentric
			Co08b	Ots12q	Omy26	Ssa11qb	
Co09	196.03	207	Co09a	Ots15p	Omy21p	Ssa07p	A, metacentric
			Co09b	Ots15q	Omy21q	Ssa07q	
Co10	239.27	346	Co10a	Ots01p	Omy04p	Ssa23	—
			Co10b	Ots27	Omy13q	Ssa06q	
Co11	280.26	419	Co11a	Ots01q	Omy23	Ssa01qa	—
			Co11b	Ots29	Omy15p	Ssa29	
Co12	215.12	286	Co12a	Ots05p	Omy08p	Ssa15qa	—
			Co12b	Ots34**	Omy10q	Ssa08q	
Co13	288.32	350	Co13a	Ots05q	Omy05q	Ssa10qa	—
			Co13b	Ots23	Omy02p	Ssa05q	
Co14	270.54	356	Co14a	Ots08p	Omy25p	Ssa09qa	—
			Co14b	Ots31	Omy14p	Ssa14qb	
Co15	282.89	355	Co15a	Ots08q	Omy25q (Omy29)	Ssa09qb	—
			Co15b	Ots13q	Omy27	Ssa20qb	
Co16	211.31	220	Co16a	Ots10p	Omy09p	Ssa18qb	—
			Co16b	Ots14p	Omy18p	Ssa16qb	
Co17	181.92	292	Co17a	Ots13p	Omy18q	Ssa27	—
			Co17b	Ots16q	Omy09q	Ssa15qb	
Co18	209.02	323	Co18a	Ots14q	Omy24	Ssa09qc	—
			Co18b	Ots16p	Omy11p	Ssa19qa	
Co19	217.65	315	Co19a	Ots17	Omy15q	Ssa17qa	—
			Co19b	Ots21	Omy14q	Ssa05p	
Co20	203.33	247	Co20a	Ots24	Omy16p	Ssa19qb	—
			Co20b	Ots32	Omy13p	Ssa12qa^†^	
Co21	130.25	184	Co21	Ots18	Omy04q	Ssa06p	C, acrocentric
Co22	194.97	243	Co22	Ots19	Omy02q	Ssa10qb	C, acrocentric
Co23	212.27	213	Co23	Ots20	Omy05p	Ssa01qb	C, acrocentric
Co24	127.18	187	Co24	Ots22	Omy16q	Ssa13qa	C, acrocentric
Co25	171.35	196	Co25	Ots25	Omy20p+q	SSa08p*** & Ssa28	A, acrocentric
Co26	184.21	225	Co26	Ots26	Omy22	Ssa21	A, acrocentric
Co27	164.56	198	Co27	Ots28	Omy28	Ssa03p	B, acrocentric
Co28	169.82	214	Co28	Ots30	Omy10p	Ssa04q	C, acrocentric
Co29	173.74	181	Co29	Ots33	OmySex	Ssa11qa	B, acrocentric
Co30	189.15	218	Co30	Ots10q	Omy08q	Ssa14qa	—
Total	6596.7	8681					

Linkage groups (Co) were randomly assigned numbers, and arm names are given as “a” and “b.” Homologous arms in Chinook salmon, rainbow trout, and Atlantic salmon are based on chromosome names for each species (Ots, Omy, and Ssa, respectively), with known orientations (p is the short arm, q is the long arm).

* and ** denote inferred relationship. There were no markers in common between Co07a and Ots11p(*), and there are markers in common between Co12b and Ots34/Ots11p(**).

*** A chromosomal arm that is composed entirely of ribosomal DNA.

The final column designates chromosomal rearrangements conserved across species; letter corresponds to phylogenetic placement in Figure 5.

† Incompletely resolved relationships between Atlantic salmon and rainbow trout according to published studies.

# might include a section of Ssa19qa.

Diploid individuals were only genotyped at nonduplicated loci. Sequences from all diploid individuals were aligned to the nonduplicated loci identified in the final reference database using BOWTIE by allowing a maximum of three nucleotide mismatches per locus. Subsequently, polymorphic loci were identified in each diploid family using STACKS, and genotypes at these loci were determined when alleles were sequenced with a depth greater than 10× per individual.

STACKS uses a maximum likelihood statistical model to identify sequence polymorphisms and determine individual genotypes (Catchen *et al.* 2011, 2013). This approach can be biased toward heterozygous genotypes when sequence depths differ between the two alleles. To correct this bias against heterozygous genotypes, genotypes were corrected after running STACKS with the Python script developed by Brieuc *et al.* (2014). Specifically, individuals were determined as heterozygotes at a locus if both alleles had a depth of more than two and the total read depth was 10× or greater.

### Linkage mapping

Linkage maps in all haploid and diploid families were constructed using software for genetic mapping, ONEMAP 2.0-3 (Margarido *et al.* 2007), implemented in R version 3.0.2 (R Development Core Team 2013). Because coho salmon have 30 chromosome pairs (Phillips and Ráb 2001), each mapping family was expected to have at least 30 linkage groups. Linkage groups were named “Co,” following the convention used in mapping studies in salmonids; this practice uses abbreviated common names for groups that are not yet anchored to chromosomes (Danzmann *et al.* 2005; Naish *et al.* 2013). RAD loci with 20% or less missing values among individuals within a family were used for linkage analyses, and these loci were assigned to linkage groups in each family separately using a minimum log of odd ratio (LOD) score of 4.0 and a maximum recombination fraction of 0.25. The LOD score was subsequently increased by 1.0 until the number of linkage groups reached 30 or higher. An integrated haploid map was first constructed from the two haploid families using MERGEMAP (Wu *et al.* 2011) because genotypes at duplicated loci were only determined in these families. This integrated haploid map was later used to examine the distribution of duplicated loci across all linkage groups and identify linkage groups involved in recent or ongoing homeologous pairing.

Recombination rates in male salmonids tend to be lower than those observed for females (Sakamoto *et al.* 2000; Ostberg *et al.* 2013), but these differences tend to decrease with high marker density and genome coverage (Rexroad *et al.* 2008; Lien *et al.* 2011). We used the female meiosis from the three diploid families to estimate marker order in these crosses. The data from all haploid and diploid female parents were then combined to calculate an integrated female haploid/diploid map using MERGEMAP.

Ordering markers in the diploid male map was computationally difficult, potentially due to reduced recombination and occasional tetrasomic inheritance in males (Allendorf and Danzmann 1997). Therefore, information from the integrated female map constructed with haploid and diploid mothers was used to infer the order and map the male meiosis in the three diploid families (diploid family 1, 2, 3). Polymorphic loci in common between the male parents and the integrated female map, as well as the Y-linked growth hormone pseudogene and sex-determining gene, sdY, were grouped using a log of odd ratio (LOD) score of 4.0 and a maximum recombination fraction of 0.25 using ONEMAP. The LOD score was subsequently increased by 1.0 until the number of linkage groups reached 30 or higher. Grouped loci were then ordered based on the known order on the integrated female map using the *make.seq* and *map* functions implemented in ONEMAP. The position of a Y-linked growth hormone pseudogene and sex-determining gene, sdY, on the male map was estimated in the two diploid families (diploid family 1 and 2) using the *try.seq* and *map* functions implemented in ONEMAP. The data from all diploid male parents were then combined to calculate an integrated male map using MERGEMAP.

### Comparative mapping with Chinook salmon and comparison with other salmonid species

The reference database for coho salmon containing duplicated and nonduplicated RAD loci was aligned to the 54,937 filtered RAD loci identified in Chinook salmon (Brieuc *et al.* 2014) using BOWTIE, allowing no more than three nucleotide mismatches per locus. Homologies between Chinook and coho salmon were determined by examining the chromosomal arm locations of shared loci between the two species. Putative centromere positions on coho linkage groups were estimated based on markers mapped in the gynogenetic diploid families in Chinook salmon (Brieuc *et al.* 2014). The order of mapped loci between the Chinook and coho salmon map was compared to determine if marker orders for chromosomes or chromosomal arms between the species were conserved. Finally, homologies identified between Chinook and coho salmon were used to infer homologies across coho salmon, rainbow trout, and Atlantic salmon using molecular markers in common between published maps (Phillips *et al.* 2009; Lien *et al.* 2011; Miller *et al.* 2012; Brieuc *et al.* 2014).

### Homeologous relationships and the distribution of duplicated loci across genomes

As we pointed out, two categories of duplicated loci were identified in this study, where one of the paralogs was polymorphic (OPP) or both paralogs were polymorphic (BPP). Duplicated loci with both paralogs polymorphic (BPP) were used to infer homeologous linkage groups because both paralogs could be mapped. The positions of duplicated loci were subsequently examined on the integrated haploid map to determine whether there was a bias in the distribution of these loci across linkage groups. A kernel smoothing approach using a sliding window of 2 cM was used to determine whether there was a regional bias in distribution of these loci for each linkage group following methods described by Brieuc *et al.* (2014). Homeologous relationships detected in coho salmon were also compared with those identified in Chinook and Atlantic salmon (Lien *et al.* 2011; Brieuc *et al.* 2014).

## Results

### Reference database of RAD loci

A reference database comprising a unique set of RAD loci was created for the purpose of sequence alignment and identification of polymorphisms across individuals. A total of 70,037 loci were sequenced with a depth greater than 5× per individual in at least 496 individuals. These loci formed the temporary reference database of RAD loci, and they were retained for further screening. Sequence alignment using BOWTIE showed that 4075 loci did not align uniquely to themselves and likely corresponded with repeat regions; therefore, these loci were removed from the temporary database. After performing the BLAST search of the temporary reference database against itself, 2085 loci did not return a match or the best match score was not the locus itself. It was possible that these loci contained repeat sequences; therefore, these loci were also removed from the temporary reference database. Sequences from the haploid individuals were aligned to the reference database using BOWTIE; 3706 loci from the haploid individuals aligned to several other loci, and these loci were thus black-listed and removed from the reference database. Additionally, 7235 loci were identified as polymorphic duplicated loci in the haploid families. The final reference database comprising 52,936 nonduplicated loci and 7235 duplicated loci, as well as the 9866 loci that were removed by screening, are given in Supporting Information, File S1.

### Linkage mapping

An initial framework map was constructed with two haploid families. Haploid family 1 and 2 had 3976 and 4048 biallelic polymorphic RAD loci, respectively, comprising a total of 6652 unique RAD loci. Among these loci, a mixture of duplicated and nonduplicated loci (3897 loci in haploid family 1; 3996 loci in haploid family 2) were successfully assigned to 30 linkage groups with a LOD score of 5.0 to 7.0. The total map length for the haploid family 1 and 2 was 3040.1 cM and 3185.5 cM, respectively. The integrated haploid map had 5377 nonduplicated markers and 1266 duplicated markers with a total map length of 3602.6 cM (File S2).

**Figure 1:**
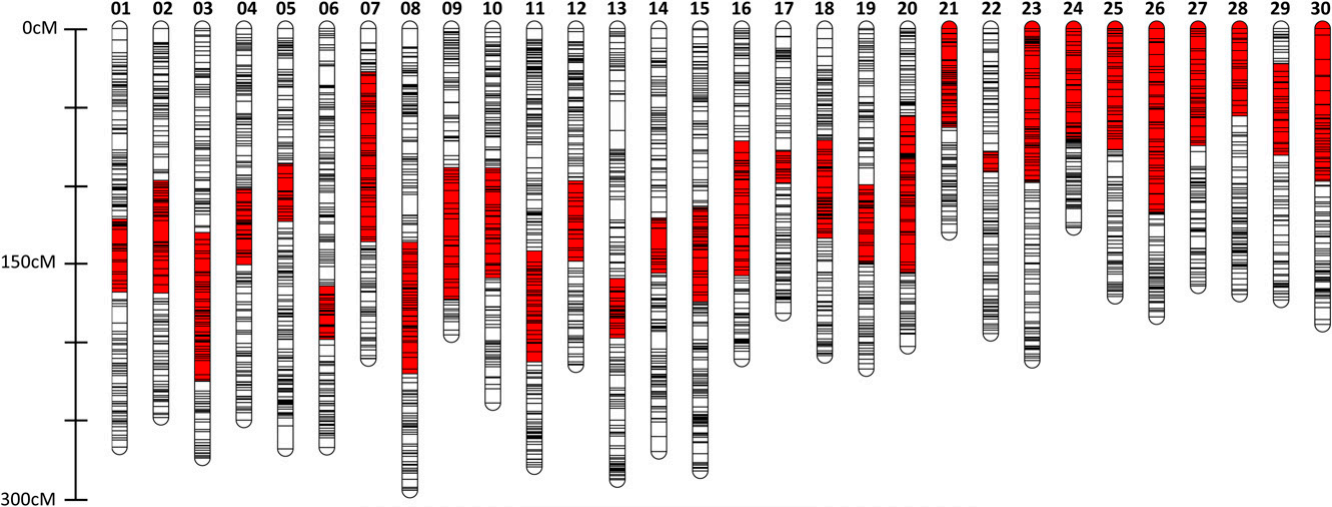
Graphical representation of 30 consensus linkage groups in haploid and diploid female coho salmon. Co01 to Co20 are metacentric, and Co21 to Co30 are acrocentric, inferred from comparative mapping. The size of linkage groups ranges from 127.18 to 295.21 cM (Kosambi), and each line corresponds to the location of one or more markers. The putative location of the centromere, estimated by comparative mapping with Chinook salmon, is represented in red.

Linkage analyses were conducted in the diploid families following the construction of the integrated haploid map. Diploid family 1, 2, and 3 had 1360, 1176, and 1931 biallelic nonduplicated loci that were polymorphic in each female parent, respectively. Among these loci, a set of loci (1214 loci in diploid family 1; 1138 loci in diploid family 2; 1765 loci in diploid family 3) were successfully assigned to 30 linkage groups with a LOD score of 4.0 to 8.0. The total map length for the diploid family 1, 2, and 3 was 3714.1 cM, 3068.9 cM, and 5047.2 cM, respectively. Although the diploid family 3 had the largest total map length, it also had the highest number of markers mapped. Because more recombination events are captured with more markers (Liu 1998), it is not surprising that the diploid family 3 had the largest total map length. Finally, data from the haploid parents and diploid female parents were combined; an integrated haploid/diploid female map measured 6596.7 cM, and it comprised 7415 nonduplicated markers and 1266 duplicated markers (Table 1; Figure 1; File S2).

The male meioses were mapped using linkage analyses in the diploid families. Among the 8681 loci placed on the integrated haploid/diploid female map, diploid family 1, 2, and 3 had 846, 814, and 879 polymorphic loci in common for each male parent. Among these loci, a set of loci (792 loci in diploid family 1; 790 loci in diploid family 2; 851 loci in diploid family 3), as well as the Y-linked growth hormone pseudogene and sex-determining gene, sdY, were successfully assigned to 30 linkage groups with a LOD score of 4.0 to 7.0. Both the growth hormone pseudogene and sdY mapped to the telomeric region of the linkage group, Co30. All linkage groups were successfully merged, except for Co22, which was split into two linkage groups (Co22_1 and Co22_2) (File S2). The number of markers in common between the integrated male and female maps varied for each linkage group, ranging from 25 to 106 markers per linkage group (File S3). The male map had a total map length of 4141.76 cM (File S3).

**Figure 2:**
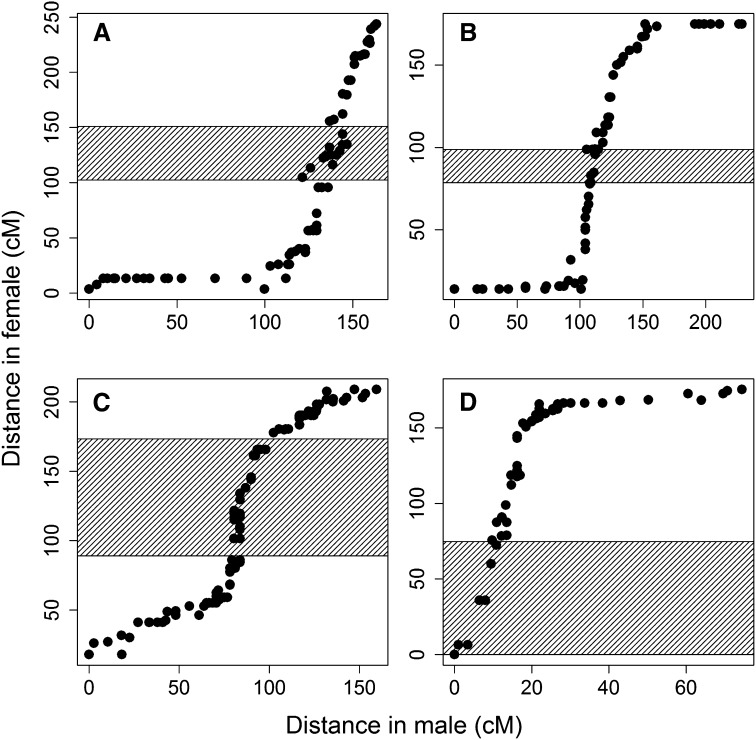
Comparison of map distances between common markers mapped in male and female coho salmon. Linkage groups Co04 (A), Co17 (B), Co18 (C), and Co26 (D) are given as examples. The putative region containing the centromere is represented by the cross-hatched area. All linkage groups are presented in File S4.

The comparison between the male and female linkage groups reflected different recombination patterns between the sexes (Figure 2; File S4). Although telomeres were not mapped in males due to a lack of duplicated markers, many male linkage groups were expanded in size toward the terminal regions relative to the female, as seen by the increased distance in these regions reflecting more recombination events. Such patterns were particularly prominent for several linkage groups (Co02, Co04, Co05, Co08–Co10, Co13–Co15, Co17–Co19, Co21–Co29). Although qualitative, there was also evidence of suppressed recombination around the region containing the centromere in the male map compared with female integrated map for all linkage groups. The male map had reduced distance in these regions compared to the female map.

### Comparative mapping with Chinook salmon and comparisons with other salmonid species

We mapped 664 RAD loci in coho salmon that had been previously placed on the Chinook salmon map, which permitted the identification of homologous chromosomal arms between the two species (Table 1). On the basis of this comparison, we also identified the putative locations of centromeres within coho salmon linkage groups (Figure 1). Two homologous relationships between the species were inferred. An arm of the linkage group Co07a had three markers that mapped to Ots34 in Chinook salmon. However, Co12b had six markers that mapped to Ots34 and three markers that mapped to Ots11p. Ots11p and Ots34 are likely involved in recent or ongoing homeologous pairing in Chinook salmon (Brieuc *et al.* 2014); therefore, it was not surprising that loci on Ots34 mapped to both homologous arms in coho salmon. In this case, we assumed that Co07a was homologous to Ots11p, and Co12b was homologous to Ots34 for reasons given in the *Discussion*.

Comparative mapping with Chinook salmon permitted inference of the structure of coho linkage groups. Twenty linkage groups in coho salmon corresponded to putative bi-armed metacentric chromosomes, and 10 linkage groups corresponded to putative uni-armed acrocentric chromosomes (Figure 1). These inferred structures are in agreement with the known chromosome structures in coho salmon (Phillips and Ráb 2001). The short (p) arm of an acrocentric chromosome is usually uncharacterized in mapping studies because there are often insufficient markers describing this region (Brieuc *et al.* 2014). In this study, we identified the small arm for two putative acrocentric chromosomes (Co22, Co29) through comparative mapping with Chinook salmon.

Comparative mapping between the Chinook and coho salmon maps also provided information on chromosomal arrangements that are shared between the two species. Eighteen chromosomes are conserved between the species (Table 1); specifically, nine metacentric chromosomes and nine acrocentric chromosomes were conserved between the species. The remaining chromosome structures likely support independent Robertsonian rearrangements that occurred after descent from a common ancestor.

Five metacentric linkage groups in coho salmon (Co10–Co14) consist of one acrocentric chromosome and one arm from a metacentric chromosome in Chinook salmon. Four metacentric linkage groups in coho salmon (Co15–Co18) comprise arms that are found in two separate metacentric chromosome pairs in Chinook salmon. Two metacentric linkage groups (Co19, Co20) comprise two acrocentric chromosome pairs in Chinook salmon. Finally, one acrocentric linkage group (Co30) corresponds to an arm that is a part of a metacentric chromosome pair in Chinook salmon.

The orders of the RAD loci on the Chinook and coho salmon maps were compared across each linkage group or for each chromosome arm to determine whether any chromosomal inversions occurred after divergence between the species. There was a strong linear relationship among mapped loci for all the linkage groups or arms (File S5), suggesting that marker orders were conserved for all chromosomes or chromosomal arms. Such analyses provide additional evidence for the occurrence of centrometric inversion in Omy20 in rainbow trout after divergence between rainbow trout and Chinook/coho salmon, and this chromosomal inversion may be exclusive to rainbow trout (Naish *et al.* 2013; Ostberg *et al.* 2013; Brieuc *et al.* 2014).

The homologies we observed between Chinook and coho salmon permitted alignment of coho linkage groups to those of rainbow trout and the Atlantic salmon, and the results are summarized in Table 1. Three acrocentric and eight metacentric chromosomes were conserved among coho salmon, Chinook salmon, and rainbow trout. Comparison between the *Oncorhynchus* species and the Atlantic salmon revealed that one metacentric chromosome and one acrocentric chromosome were conserved across all compared species.

### Homeologous relationships and the distribution of duplicated loci across linkage groups

**Figure 3:**
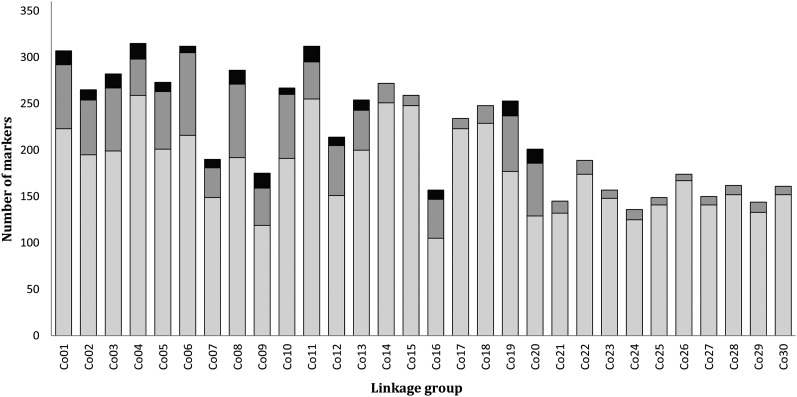
Number of markers and distribution of duplicated markers across each coho salmon linkage group. Nonduplicated loci are represented in light gray. Duplicated loci are represented in dark gray (loci with only one paralog polymorphic) or black (both paralogs polymorphic).

**Figure 4:**
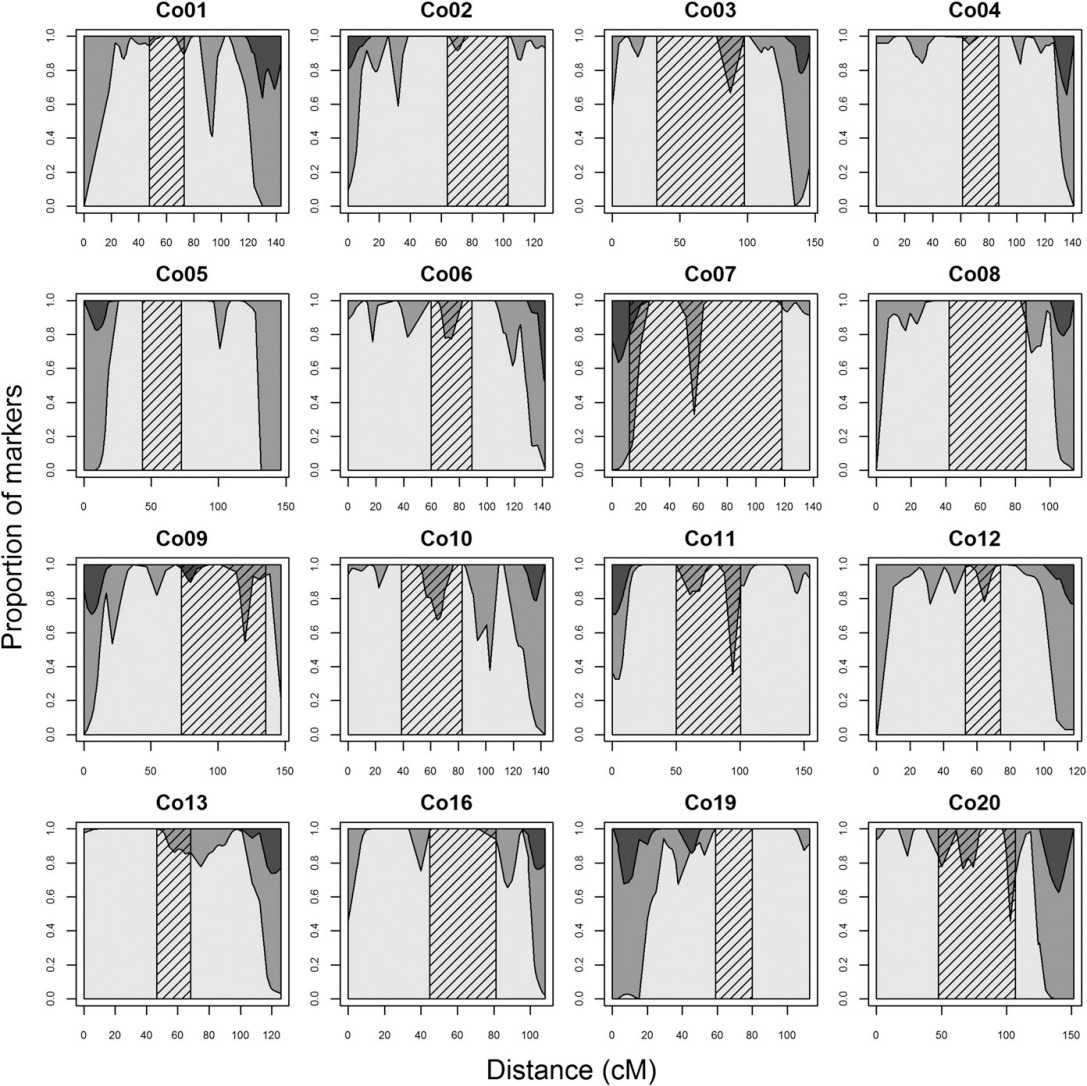
Distribution of duplicated and nonduplicated loci along the 16 linkage groups with a high proportion of duplicated loci. Nonduplicated loci are represented in light gray. Duplicated loci are represented in dark gray (loci with one paralog polymorphic) or in black (loci with both paralogs polymorphic). The putative region containing the centromere is represented by the cross-hatched area.

The identification of linkage groups involved in homeologous pairing, as well as the localization of duplicated loci across individual linkage groups, was examined using the integrated haploid female map. A total of 1266 duplicated loci (1066 OPP and 200 BPP) were placed on this map. These loci were not distributed evenly among the linkage groups (χ^2^ test for uniform distribution across linkage groups, after correction for the number of markers per linkage group: p-value ∼0, *df* = 29); 87.0% of the duplicated loci were located on 16 linkage groups (Figure 3). There was a bias in distribution of these loci along the 16 linkage groups; duplicated loci were mostly found in the distal regions of all 16 linkage groups (Figure 4). Homeologies were identified between putative chromosome arms using marker pairs in which both paralogs were polymorphic (Table 2). All eight homeologous arm pairs with a high retention of duplicated loci detected in coho salmon were also observed in Chinook salmon, and four homeologous arm pairs were conserved in Atlantic salmon (Table 2). All of these chromosome arms, likely involved in recent or ongoing homeologous pairing, involved at least one metacentric chromosome ancestral to the divergence between Pacific salmon species.

**Table 2:** Homeologous chromosome arm pairs identified in coho salmon and the number of marker pairs supporting the homeologous relationship

Homeology in Coho Salmon	Number of Marker Pairs Supporting Homeolog Pairings	Homeology in Chinook Salmon (Brieuc *et al.* 2014)	Homeology in Rainbow Trout (Phillips *et al.* 2006)	Homeology in Atlantic Salmon (Lien *et al.* 2011)	Known Phylogenetic Placement of Metacentric Arrangement
Co01b–Co20b	15	Ots02q–Ots32	Omy17q^‡^–Omy13p	Ssa02q–Ssa12qa^†^	B
Co02a–Co13b	11	Ots03p–Ots23	Omy03p–Omy02p	Ssa02p–Ssa05q	B*
Co03b–Co08b	15	Ots04q–Ots12q	Omy06q–Omy26	Ssa26–Ssa11qa^†^	B, C
Co04b–Co11a	17	Ots06q–Ots01q	Omy01q–Omy23	Ssa18qa–Ssa01qa^†^	B
Co05a–Co16b	10	Ots07p–Ots14p	Omy07p–Omy18p	Ssa17qa–Ssa16qb^†^	B
Co06b–Co10b	7	Ots09q–Ots27	Omy12q–Omy13q	Ssa03q–Ssa06p	B*
Co07a–Co12b	9	Ots11p–Ots34	Omy19p–Omy10q	Ssa04p–Ssa08q	B*
Co09a–Co19a	16	Ots15p–Ots17	Omy21p–Omy15q	Ssa07p–Ssa17qa	A

**Figure 5:**
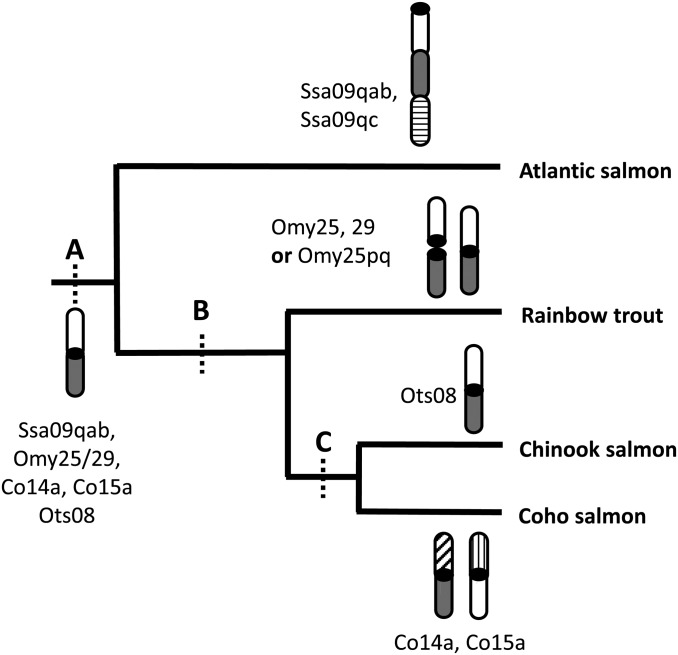
Phylogenetic tree showing the orientation of homologous arms Ssa09qab, Omy25/29, Co14a, Co15a, and Ots08 in Atlantic salmon, rainbow trout, coho salmon, and Chinook salmon, respectively. Chromosomal rearrangements and homeologous relationships conserved across species at phylogenetic nodes A, B, C are summarized in Table 1 and Table 2.

Corresponding known homeologous relationships in Chinook salmon, rainbow trout, and Atlantic salmon are shown. The final column designates known conservation of metacentric chromosome with high frequency of duplicated markers. Letter corresponds to phylogenetic placement in Figure 5.

* Possible earlier chromosomal arrangement (A) and subsequent rearrangement.

† Homeologous relationships with little or no support in Atlantic salmon (Lien *et al.* 2011).

‡ We have corrected the homeologous relationship between Omy17q and Omy13p; evidence suggests that this relationship was incorrectly reported as being between Omy17p and Omy13p in previous studies.

## Discussion

Here, we aimed to examine the relationship between chromosomal evolution and retention of duplicated regions within the genus *Oncorhynchus*, and between this genus and *Salmo*, by deriving a linkage map for coho salmon and comparing this map with that of Chinook salmon, rainbow trout, and Atlantic salmon. Thirty linkage groups including 20 putative metacentric and 10 putative acrocentric chromosomes were described across two haploid and three diploid families. Chromosomal rearrangements were identified by comparing homologous arms between coho salmon, Chinook salmon, rainbow trout, and Atlantic salmon. Results confirmed the conservation of at least one metacentric chromosome between *Oncorhynchus* and *Salmo* (Co09) and seven metacentric chromosomes across the genus *Oncorhynchus* (Co01–Co07) (Naish *et al.* 2013; Phillips *et al.* 2013; Brieuc *et al.* 2014), and detected a polymorphism in another across coho and Chinook salmon and rainbow trout (Co14 and Co15). Another metacentric chromosome was detected as ancestral to coho and Chinook salmon only (Co08). The placement of 1266 duplicated loci on the consensus haploid map of 6643 markers revealed that these loci were not evenly distributed across all linkage groups, supporting a previous finding in Chinook salmon (Brieuc *et al.* 2014), namely, that homeologous pairs diverged from each other at different rates following the whole genome duplication event. Regions of the genome with polymorphic duplicated markers were found on the same eight pairs of homologous chromosome arms (16 arms in total) across coho and Chinook salmon. Each of the eight pairs of chromosomes likely involved in ongoing or recent homeologous pairing included at least one of the ancestral metacentric chromosomes that are conserved between the two species. The other chromosome arm involved in the pairing may be part of either an acrocentric chromosome or a metacentric chromosome. The data suggest that Robertsonian rearrangements that result in metacentric chromosome formation prior to the diversification of homeologous pairs might partly explain the uneven retention of duplicated regions across the genome, at least within Pacific salmon.

The consensus male map, constructed with three diploid families, was significantly smaller (4141.7 cM) than the consensus female map (6596.7 cM) constructed with two haploid families and three diploid families. There are three main reasons that might explain the reduced map size in the male compared with the female map. First, the difference could simply be a function of more markers being placed on the female map (8681 for the female and 2043 for the male), because map size tends to increase when more markers are added (Liu 1998). Second, some male linkage groups only represented a portion of those in females; for example, in three metacentric chromosomes (Co07, Co09, Co13) only one arm and a region containing the centromere were mapped, the duplicated markers were not mapped in males. Third, recombination in males is suppressed relative to females, and male maps in salmonids often tend to be smaller. Comparisons between the consensus male map and the consensus (haploid and diploid) female map indicated that recombination in the males was suppressed around the region containing the centromere in some linkage groups, whereas recombination in females seemed suppressed toward telomeric regions.

Accurate identification of chromosome structure in coho salmon relied on aligning homologous chromosome arms with Chinook salmon. In addition, regions containing the centromere were inferred through comparative mapping between coho and Chinook salmon as no gynogenetic diploid families were used for this study to identify the exact location of the centromere. Homology of Co07a and Co12b with Chinook salmon chromosome arms Ots11p and Ots34 was not completely resolved. These arms are homeologous to each other within both species, and markers on Ots34 mapped to both Co07a and Co12b in coho salmon. We inferred that Co07a was homologous to Ots11p, as the arm Co07a is part of a metacentric chromosome that is conserved in Chinook salmon (Ots11) and rainbow trout (Omy19) (Table 1). In fact, Naish *et al.* (2013) speculate that these are conserved across the genus because Chinook salmon and rainbow trout are distantly related. In contrast, the metacentric chromosome Co12 is not conserved in Chinook salmon and rainbow trout. We assumed these structures in the subsequent discussion.

### Coho salmon map coverage, size, and differences in sex-specific recombination

The coho salmon linkage map constructed in this study has 8681 markers, spanning all predicted 30 linkage groups. This coverage is comparable with recently published maps across a number of salmon species (Lien *et al.* 2011; Everett *et al.* 2012; Miller *et al.* 2012; Brieuc *et al.* 2014; Gonen *et al.* 2014). We observed different map sizes in the consensus female maps; the map constructed with combined haploid and diploid families had a size of 6596.7 cM, which is significantly larger than the coho map created with haploids alone (3602.6 cM). There are several reasons that might explain the differences such as nonrandom missing values (Jorgenson *et al.* 2005), genotyping errors (Hackett and Broadfoot 2003), and numbers of markers mapped. Potential bias against heterozygotes in RAD sequencing (Brieuc *et al.* 2014) may also partly explain the inflated map distances in the haploid/diploid map, especially because the size of the map created with only haploid families in this study was much smaller.

Male salmonids are the heterogametic sex (Allendorf and Thorgaard 1984). In this study, both the Y-linked growth hormone pseudogene and sex-determining gene, sdY, mapped to the telomeric region on the acrocentric chromosome, Co30. This finding is in agreement with previous findings (Phillips *et al.* 2005). Mapping has shown that the sex chromosome is not conserved across the species, and that a small male-specific region including the sex determining gene has been repeatedly transposed to different chromosomes in different salmon species (Phillips *et al.* 2001; Woram *et al.* 2003; Yano *et al.* 2012) and can be polymorphic within a species (Eisbrenner *et al.* 2014).

Our results showed that suppressed recombination around the region containing the centromere in males was widely apparent, whereas higher recombination was observed in telomeric regions for some male linkage groups relative to female. The results are in agreement with a number of studies performed on other salmonid species (Sakamoto *et al.* 2000; Moen *et al.* 2008; Rexroad *et al.* 2008; Lien *et al.* 2011). Male recombination rate in telomeric regions of a subset of rainbow trout linkage groups has been shown to be higher than that of females, but lower in centromeric regions (Sakamoto *et al.* 2000). Such different recombination patterns might be partly explained by occasional multivalent formation during male meiosis, in which crossovers between homeologous chromosomes are increased in the telomeric regions while crossovers between homologous chromosomes are hindered in the centromeric regions through structural constraints (Sakamoto *et al.* 2000). In our study, however, suppressed recombination around the centromere and increased recombination in telomeric regions were apparent for most male linkage groups, including the ones not likely involved in homeologous pairing. Some studies have also found notable clustering of markers in centromeric regions for many male linkage groups (Nichols *et al.* 2003; Lien *et al.* 2011; Miller *et al.* 2012). In Atlantic salmon, high marker densities were also involved in regions close to the centromere for male linkage groups with a lower frequency of duplicated markers (Lien *et al.* 2011). Recombination rates are known to differ between the sexes in a wide range of species (Lenormand and Dutheil 2005); although homeologous chromosome pairing during male meiosis could certainly account for some of the differences, the origin of the sex differences observed in this study still remains unclear.

### Comparative genome mapping

Comparative mapping provided insights into the process of chromosomal evolution occurring after the whole genome duplication event, and this is the first study that characterizes chromosomal evolution between coho and Chinook salmon. Nine metacentric and nine acrocentric chromosomes appear to be conserved between these two species. Among these conserved chromosomes, one metacentric (Co08 in coho and Ots12 in Chinook) and five acrocentric chromosomes are unique to coho and Chinook salmon, suggesting that the structure of these linkage groups is ancestral to the divergence of these species relative to rainbow trout and Atlantic salmon. The remaining linkage groups are not conserved, reflecting extensive chromosomal rearrangements since coho and Chinook salmon diverged.

Syntenic relationships between the Chinook salmon and rainbow trout maps permitted comparisons across the genus *Oncorhynchus* (Naish *et al.* 2013; Phillips *et al.* 2013). There are four acrocentric chromosomes that are conserved across all three *Oncorhynchus* species (Co26–Co29). Similarly, eight metacentric chromosomes are conserved among all three species (Co01–Co07, Co09). The results support the hypothesis of Naish *et al.* (2013) that the arm rearrangements that resulted in these metacentric chromosomes are ancestral to the divergence of the species and could be conserved across the genus *Oncorhynchus*. There is one interesting extension to these earlier observations. One metacentric chromosome in Chinook salmon, Ots08, sometimes occurs as a metacentric chromosome (Omy25p and q) or as two acrocentric arms (Omy25 and Omy 29) in rainbow trout (Figure 5). Here, we found that the homologous arms in coho salmon occur in two separate unrelated metacentric linkage groups (Co14a and Co15a) (Figure 5). In Atlantic salmon, these two arms are fused together along with a third arm to form a large acrocentric chromosome (Figure 5). Robertsonian fusions are common and can also form acrocentric chromosomes; this outcome is likely more frequent in Atlantic salmon than in Pacific salmon (Phillips and Ráb 2001). Taken together, the configurations of these particular chromosomes suggest they may have undergone recurrent fusions and fissions across species.

One metacentric and one acrocentric chromosome, Co09 and Co26, respectively, appear to be conserved across coho salmon, Chinook salmon, rainbow trout, and Atlantic salmon (Phillips *et al.* 2009; Brieuc *et al.* 2014). Our study supports the previous findings that this metacentric chromosome is likely ancestral to the divergence of the two genera, *Salmo* and *Oncorhynchus*. It also appears that this chromosome is conserved in Arctic charr and Brook charr within the genus *Salvelinus* (Timusk *et al.* 2011), both of which share a more recent common ancestor with *Oncorhynchus*. In addition, previous results provided evidence for the occurrence of centrometric inversion in Omy20 in rainbow trout following divergence between rainbow trout and Chinook/coho salmon, and this chromosomal inversion may be exclusive to rainbow trout (Naish *et al.* 2013; Ostberg *et al.* 2013; Brieuc *et al.* 2014). In the current study, marker orders are fully conserved between coho and Chinook salmon chromosome arms, Co25 and Ots25, respectively, further supporting this previous observation (File S5).

### Conservation of reduced divergence between homeologous chromosomes across species

Although our results confirm that the divergence rates of homeologs following the whole genome duplication event have not been uniform (Brieuc *et al.* 2014), the key finding of this study is that the ancestral metacentric chromosomes retain recently diverged duplicates and are the ones likely involved in recent or ongoing homeologous pairing (Co01–Co07, conserved among all three *Oncorhynchus* species; Co09, conserved among all four species). Such findings suggest that homeologies may be preferentially retained between larger metacentric chromosomes (Phillips *et al.* 2009), and the involvement of at least one metacentric chromosome provides the stability required for the formation of multivalents (Wright *et al.* 1980, 1983; Brieuc *et al.* 2014). These results support the hypothesis of Phillips *et al.* (2009), which suggested that diploidization of chromosomes not involved in homeologous parings may have occurred in the ancestral salmonid before the divergence between *Salmo* and *Oncorhynchus*. We speculate that this process also differed to some extent following the divergence of the two genera. Although the exact distribution of duplicated markers along chromosome arms in Atlantic salmon has not yet been described (Lien *et al.* 2011), only four out of eight homeologous pairings appear to share polymorphic duplicated loci between the Atlantic salmon and Chinook and coho salmon grouping (Table 2).

The implications of our findings for species divergence within the subfamily Salmoninae will become clearer once we gain a greater understanding of the role of duplicated regions in evolution. If the duplicated regions we detected have genes that permit greater flexibility for adaptation by providing the opportunity to acquire additional or novel functions (Soltis and Soltis 2000; Koop and Davidson 2008; Van De Peer *et al.* 2009; Feldman *et al.* 2012; Alexandrou *et al.* 2013; Macqueen and Johnston 2014), then retention of particular duplicated regions within certain lineages may explain their subsequent innovation and diversification. However, the physical formation of metacentric chromosomes may inhibit diploidization because of ongoing recombination; such chromosomes may continue to exhibit tetrasomic inheritance, thus becoming “evolutionary dead ends.” Whole genome sequencing of duplicated chromosome arms in rainbow trout points toward the fact that duplicated protein coding loci have simply become lost through gradual change (Berthelot *et al.* 2014); the conserved metacentric chromosomes may continue exhibiting tetrasomic inheritance and prevent functional divergence of protein coding regions. In this study, we provide preliminary evidence that the evolutionary timing of metacentric chromosome formation varied, which might have impacted the rate of diploidization across different lineages. As comparative genome sequencing of salmon species continues, comparing the rates of differentiation along certain chromosome arms between species and identifying the location of genes involved in diversification will provide insights into the role of WGD in salmon evolution. Here, we have identified chromosome arms of interest for further efforts addressing such questions.

## Conclusion

Here, we developed an extensive set of genomic resources for coho salmon: a reference database of unique RAD loci, two types of consensus female linkage maps, and a consensus male linkage map. A dense female map constructed in this study permitted alignment of linkage groups in this species with that of Chinook salmon, enabling interspecies comparisons with related salmon species. Syntenic relationships across multiple salmonid species identified in this study provided strong evidence for chromosomal rearrangements and conservation of metacentric and acrocentric chromosomes following the divergence between *Salmo* and *Oncorhynchus*. We have also identified linkage groups that recently have been or may be involved in ongoing homeologous pairing in coho salmon. Such pairings were conserved with related Pacific salmon species. Ancestral metacentric chromosomes appear to retain recently diverged duplicated regions and may be involved in ongoing homeologous pairings; such results indicate that diploidization may have been prevented or retarded in these ancestral metacentric chromosomes following the whole genome duplication event. The resources developed here will facilitate genome-wide studies in coho salmon, such as genome scans, QTL mapping, and genome-wide association studies (Naish and Hard 2008), as well as provide resources for studies concerning ecology and evolution in related salmon species.

## Supplementary Material

Supporting Information
