# Supplementary material for: Comparative Mapping Between Coho Salmon (Oncorhynchus kisutch) and Three Other Salmonids Suggests a Role for Chromosomal Rearrangements in the Retention of Duplicated Regions Following a Whole Genome Duplication Event
Source: G3 (Bethesda). 2014 Jul 21;4(9):1717–30. doi: 10.1534/g3.114.012294 (PMC4169165; doi:10.1534/g3.114.012294)
Supplement: Supporting Information [file supp_4_9_1717__index.html]

Comparative Mapping Between Coho Salmon (Oncorhynchus kisutch) and Three Other Salmonids Suggests a Role for Chromosomal Rearrangements in the Retention of Duplicated Regions Following a Whole Genome Duplication Event — Supporting Information 

# Comparative Mapping Between Coho Salmon (*Oncorhynchus kisutch*) and Three Other Salmonids Suggests a Role for Chromosomal Rearrangements in the Retention of Duplicated Regions Following a Whole Genome Duplication Event

## Supporting Information for Kodama *et al.*, 2014

**Files in this Data Supplement:**

- Supporting Information - Files S1-S5 (PDF, 1 MB)
- File S4 - Relationship among positions of mapped RAD loci in common between the sexes. (PDF, 1 MB)
- File S5 - Relationship among mapped RAD loci in coho and Chinook salmon for all the linkage groups or arms. (PDF, 821 KB)
- File S1 - Reference database of RAD loci for coho salmon. (.xls, 3.7 MB)
- File S2 - Linkage maps. (.xls, 702 KB)
- File S3 - Difference in sizes between integrated female and male coho salmon linkage groups. The map lengths are presented in centiMorgan (Kosambi). (.xlsx, 12 KB)
