# Supplementary material for: Comparative Mapping Between Coho Salmon (Oncorhynchus kisutch) and Three Other Salmonids Suggests a Role for Chromosomal Rearrangements in the Retention of Duplicated Regions Following a Whole Genome Duplication Event
Source: G3 (Bethesda). 2014 Jul 21;4(9):1717–30. doi: 10.1534/g3.114.012294 (PMC4169165; doi:10.1534/g3.114.012294)
Supplement: Supporting Information [file supp_g3.114.012294_012294SI.pdf]

G3 · Genes | Genomes | Genetics

Comparative mapping between coho salmon (*Oncorhynchus kisutch)* and three other salmonids suggests a role for chromosomal rearrangements in the retention of duplicated regions following a whole genome duplication event

Miyako Kodama[1§], Marine S O Brieuc[1], Robert H Devlin[2], Jeffrey J Hard[3] and Kerry A Naish[1 §]

[1] School of Aquatic and Fishery Sciences, University of Washington, Seattle, WA 98105, USA

[2] Fisheries and Oceans Canada, West Vancouver, British Columbia, V7K 1N6, Canada

[3] National Marine Fisheries Service, Northwest Fisheries Science Center, Seattle, WA 98112, USA

[§]Corresponding authors

Email addresses:

MK: mkodama@uw.edu

MSOB: mbrieuc@uw.edu

RHD: robert.devlin@dfo-mpo.gc.ca

JJH: Jeff.Hard@noaa.gov

KAN: knaish@uw.edu

DOI: 10.1534/g3.114.012294

File S1: Reference database of RAD loci for coho salmon

S1.1: Non-duplicated RAD loci in the reference database and corresponding sequences. Mapped markers have chromosome arm added to the name (CoXXXXX_CoXX[a or b]). Markers falling in the putative location of the centromere, estimated by comparative mapping with Chinook salmon, have the linkage group followed by u (CoXXXXX_CoXXu) where u designates arm placement unknown.

S1.2: Duplicated RAD loci in the reference baseline and corresponding sequences. The letter D was added after the marker name. Mapped markers have chromosome arm added to the name (CoXXXXX_CoXX[a or b]). Markers falling in the putative location of the centromere, estimated by comparative mapping with Chinook salmon, have the linkage group followed by u (CoXXXXX_CoXXu).

S1.3: RAD loci removed from the temporary reference database during screening and sequences.

File S2: Linkage maps

S2.1: The consensus female map constructed with two haploid families; marker distances are shown in centiMorgan (Kosambi distance).

S2.2: The consensus female map constructed with two haploid and three diploid families; details given are linkage groups, marker distances in centiMorgan (Kosambi), assignment of the marker to the Chinook salmon female map, Chinook salmon chromosomes and its position on the Chinook map.

S2.3: The consensus male map constructed with three diploid families; details given are linkage groups, marker distances in centiMorgan (Kosambi) and corresponding marker position on the consensus female map constructed with haploid and diploid female parents.

File S3: Difference in sizes between integrated female and male coho salmon linkage groups. The map lengths are presented in centiMorgan (Kosambi).

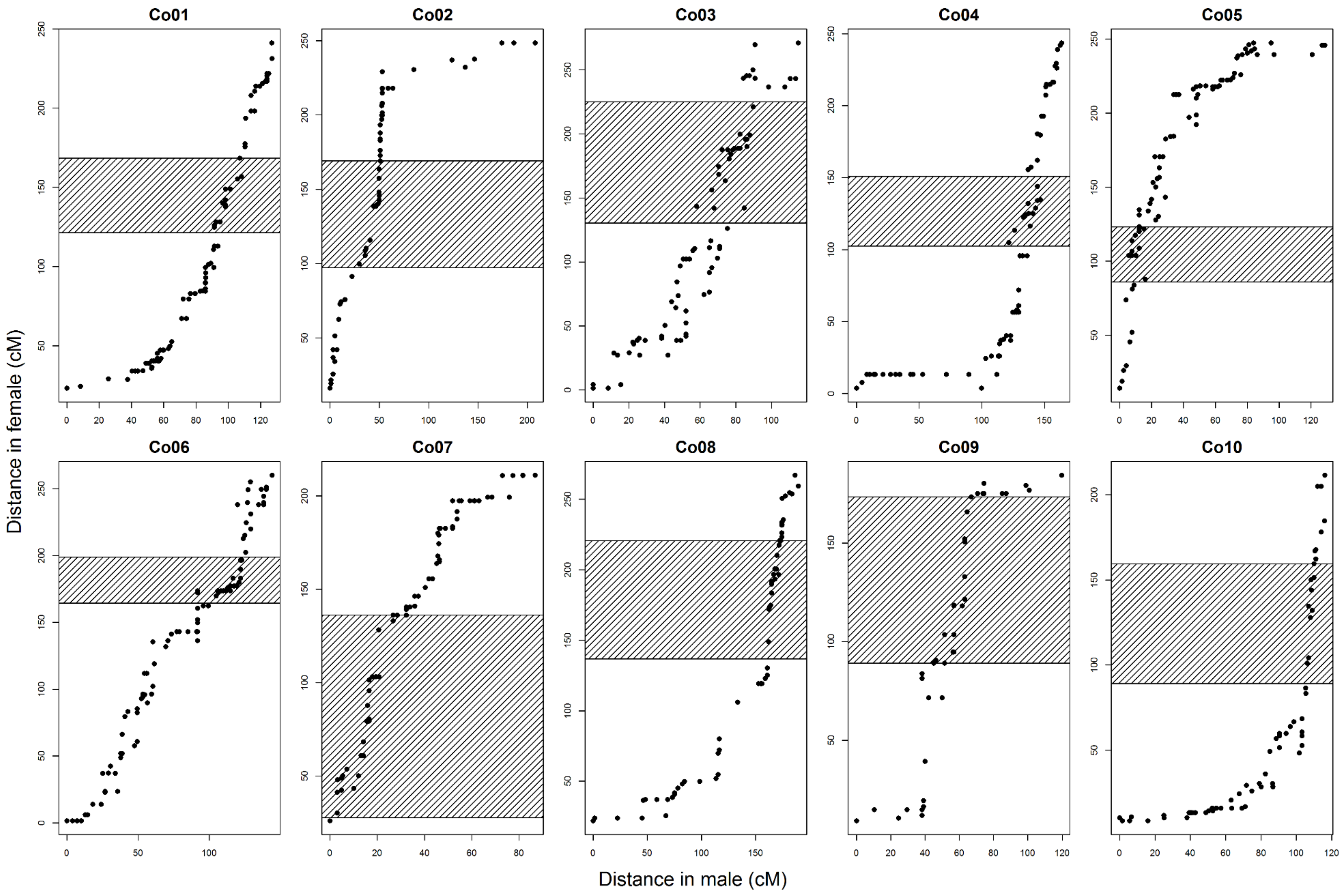
Co01
Co02
Co03
Co04
Co05
Co06
Co07
Co08
Co09
Co10
Distance in female (cM)
Distance in male (cM)

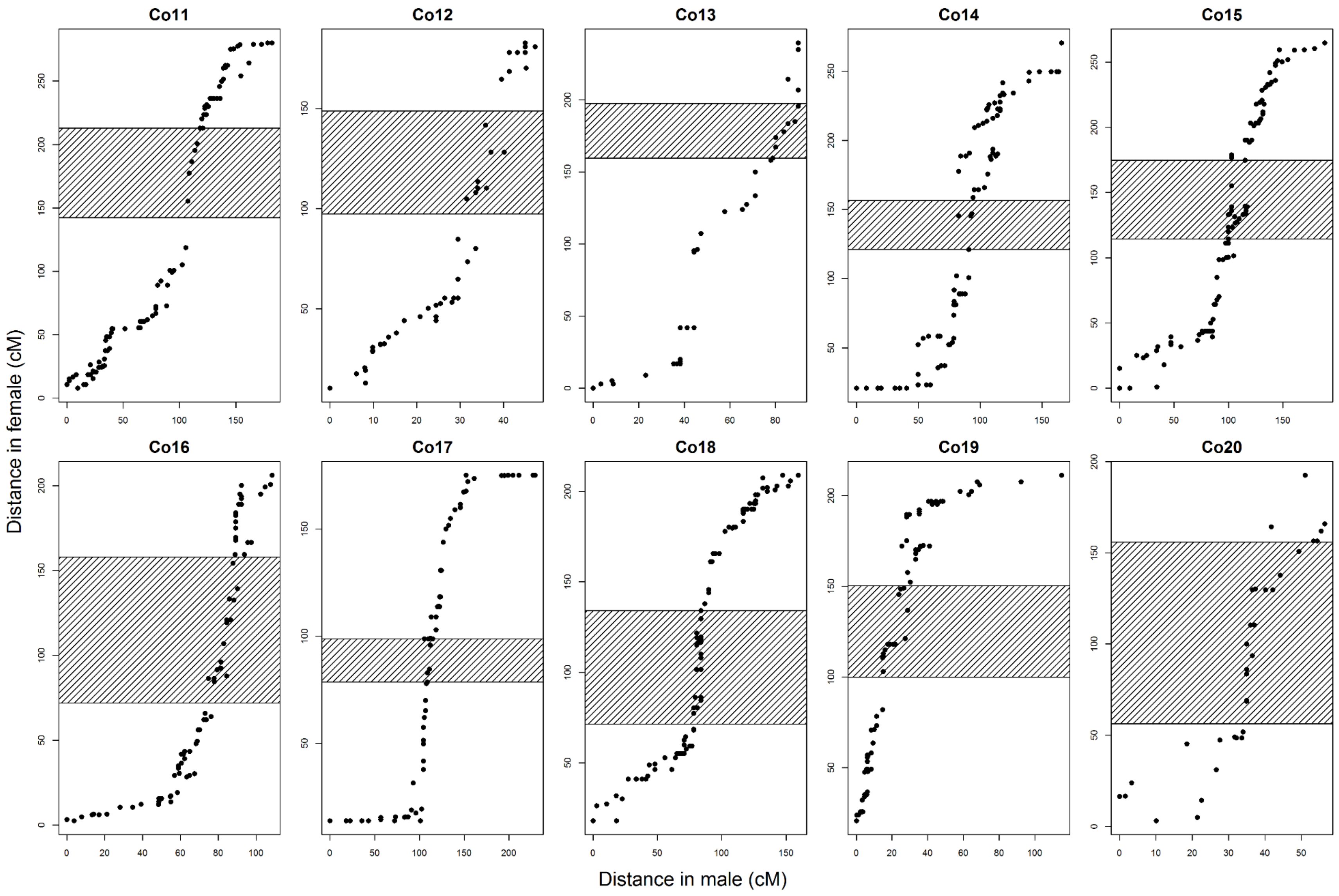
Co11
Co12
Co13
Co14
Co15
Co16
Co17
Co18
Co19
Co20
Distance in female (cM)
Distance in male (cM)

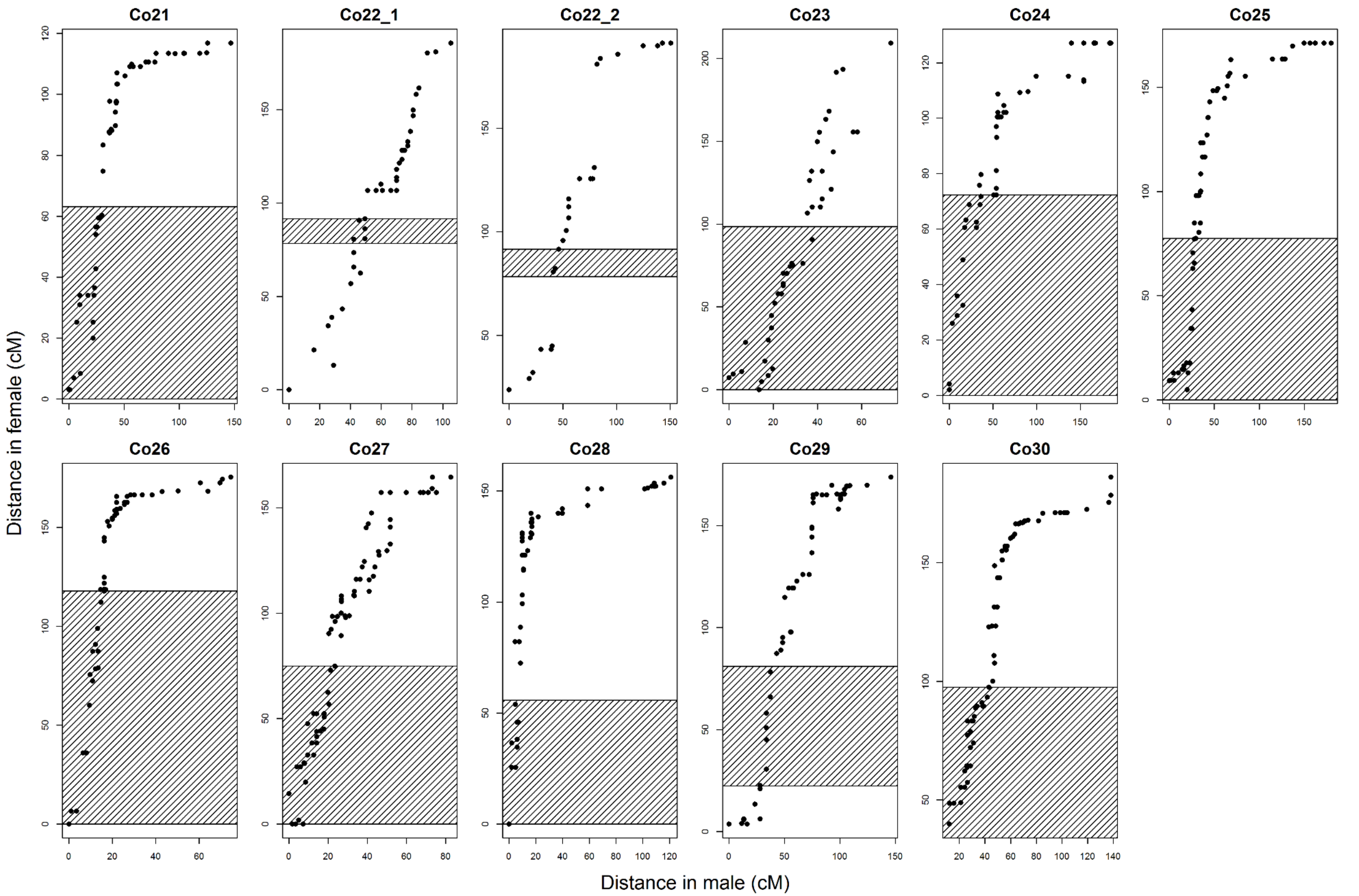
Co21
Co22_1
Co22_2
Co23
Co24
Co25
Co26
Co27
Co28
Co29
Co30
Distance in female (cM)
Distance in male (cM)

File S4: Relationship among positions of mapped RAD loci in common between the sexes. Presumed regions containing the centromere are represented by the cross-hatched area. The consensus female map constructed with haploid and diploid families (S2.2) was used for this comparison.

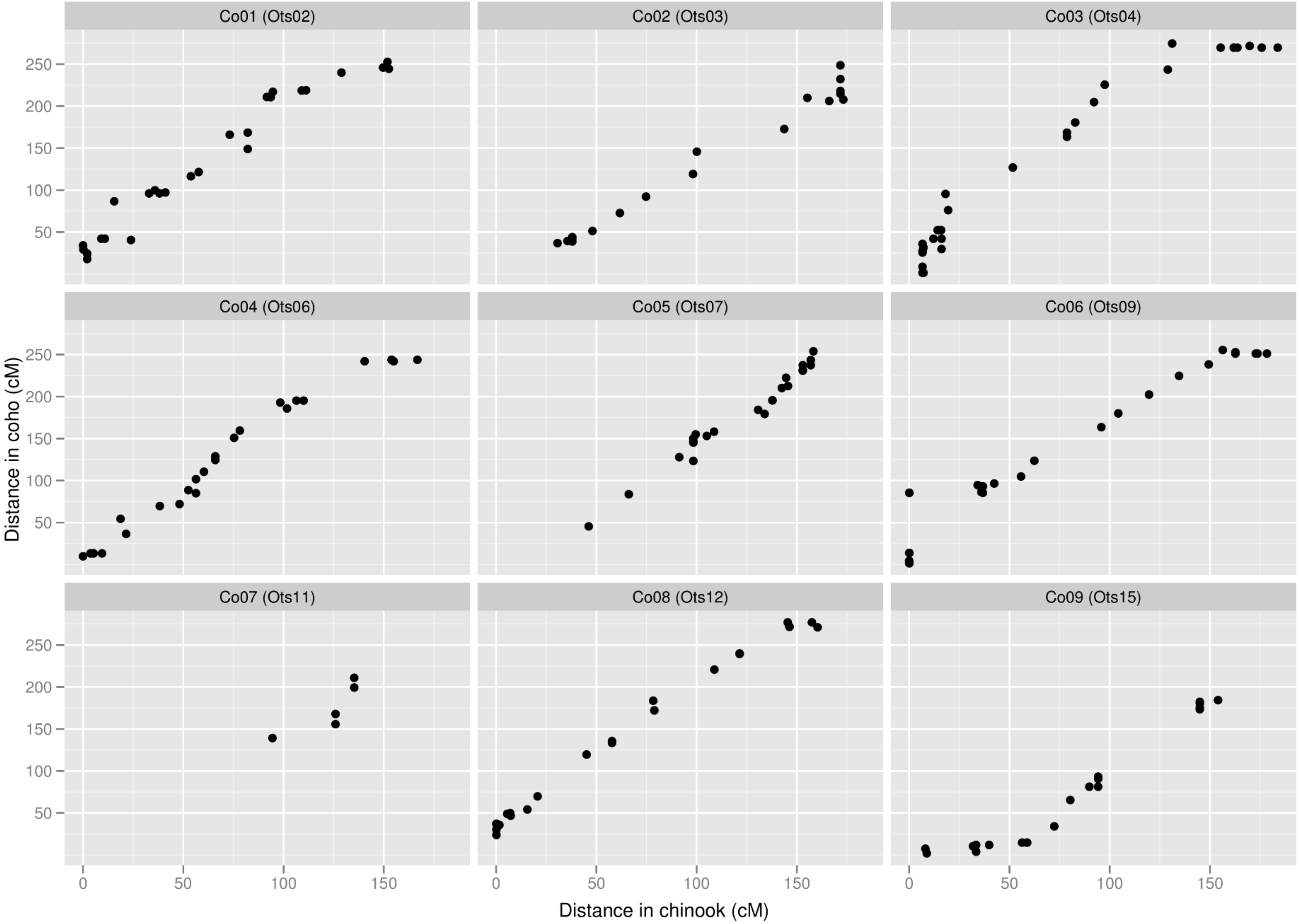
Co01 (Ots02)
Co02 (Ots03)
Co03 (Ots04)
Co04 (Ots06)
Co05 (Ots07)
Co06 (Ots09)
Co07 (Ots11)
Co08 (Ots12)
Co09 (Ots15)
Distance in coho (cM)
Distance in chinook (cM)
50
100
150
200
250
0
50
100
150

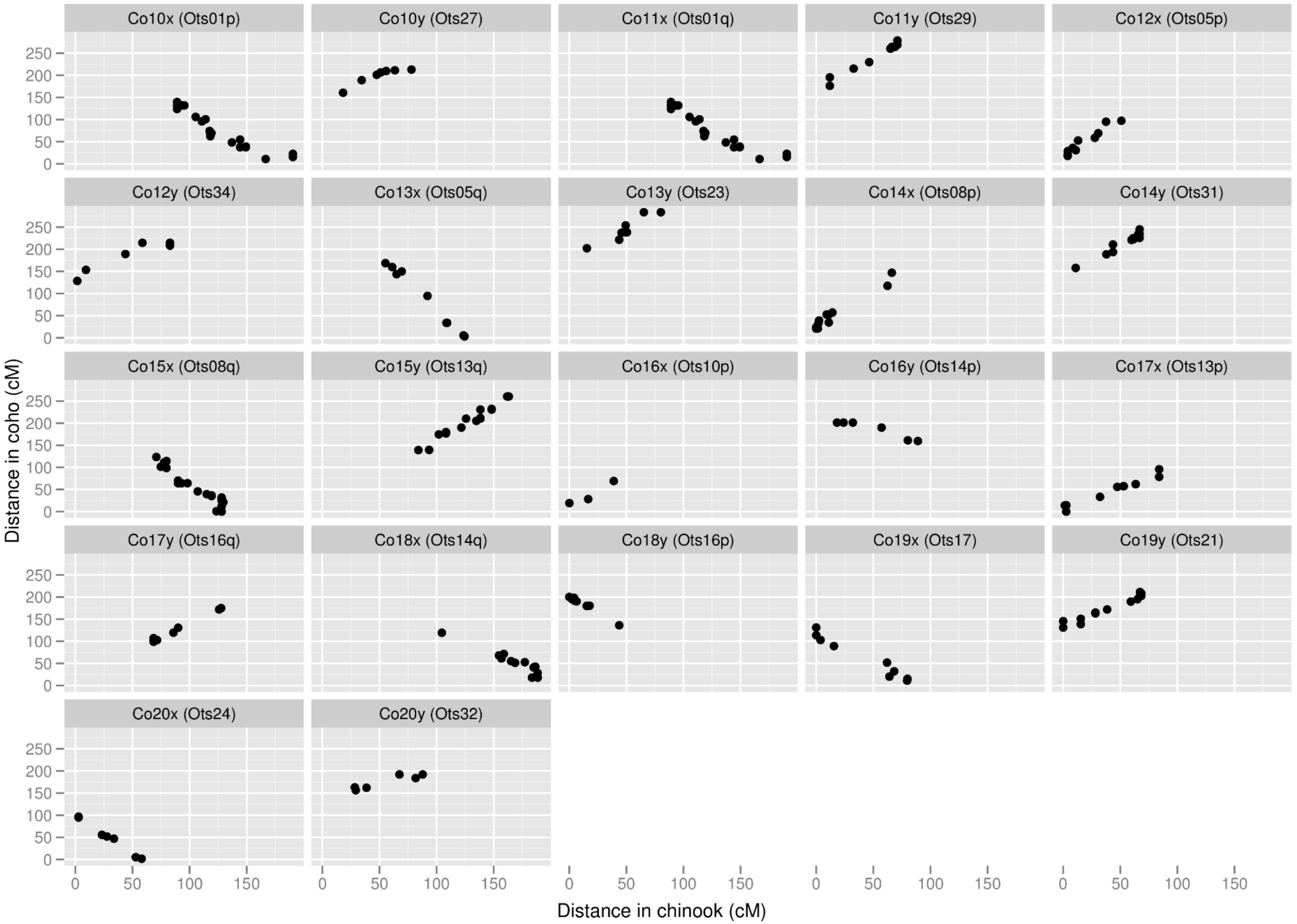

Co10x (Ots01p)
Co10y (Ots27)
Co11x (Ots01q)
Co11y (Ots29)
Co12x (Ots05p)
Co12y (Ots34)
Co13x (Ots05q)
Co13y (Ots23)
Co14x (Ots08p)
Co14y (Ots31)
Co15x (Ots08q)
Co15y (Ots13q)
Co16x (Ots10p)
Co16y (Ots14p)
Co17x (Ots13p)
Co17y (Ots16q)
Co18x (Ots14q)
Co18y (Ots16p)
Co19x (Ots17)
Co19y (Ots21)
Co20x (Ots24)
Co20y (Ots32)
Distance in coho (cM)
Distance in chinook (cM)
0
50
100
150
200
250

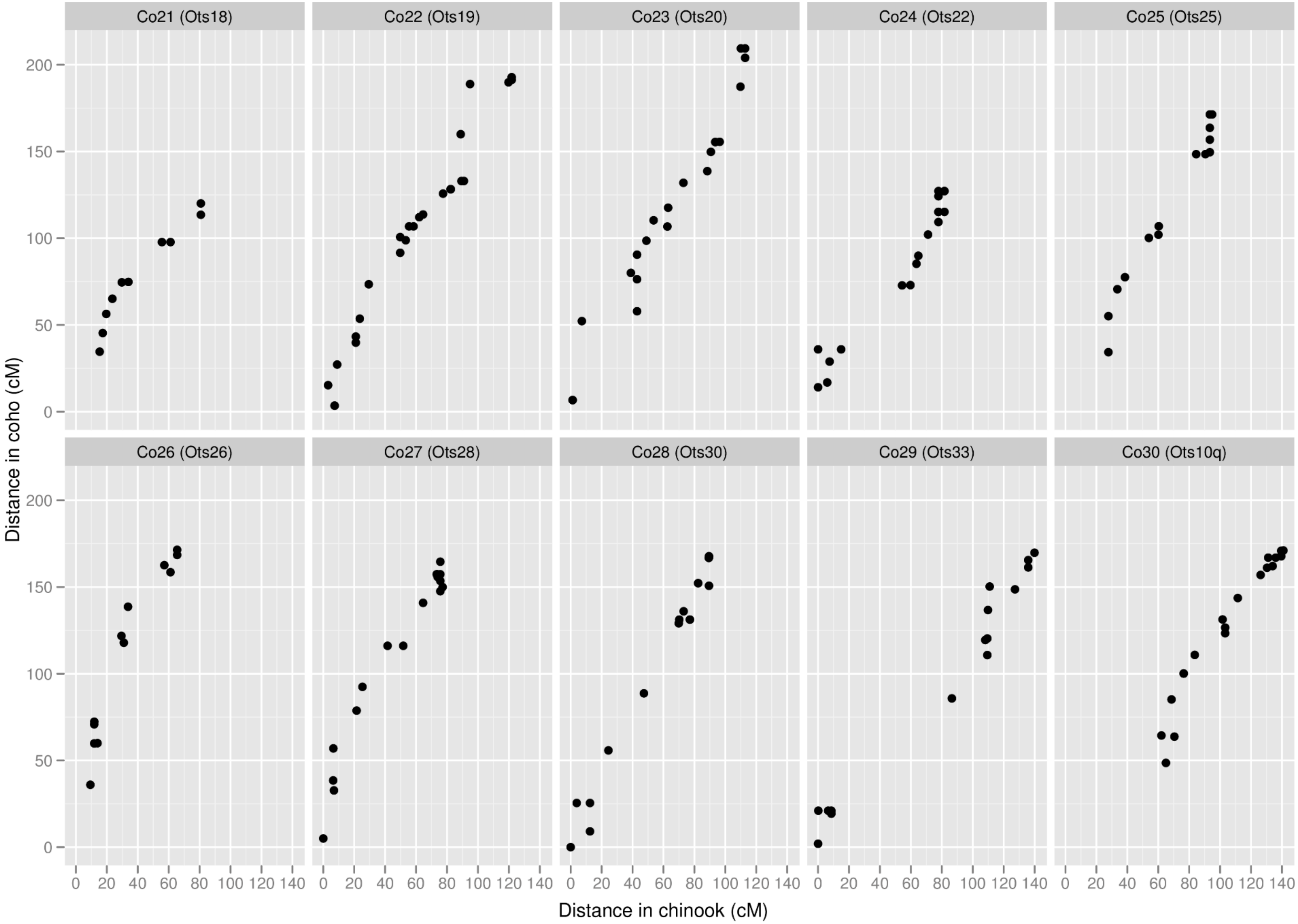

Co21 (Ots18)
Co22 (Ots19)
Co23 (Ots20)
Co24 (Ots22)
Co25 (Ots25)
Co26 (Ots26)
Co27 (Ots28)
Co28 (Ots30)
Co29 (Ots33)
Co30 (Ots10q)
Distance in coho (cM)
Distance in chinook (cM)
0
50
100
150
200
0 20 40 60 80 100 120 140

File S5: Relationship among mapped RAD loci in coho and Chinook salmon for all the linkage groups or arms.
