# Supplementary material for: Comparative Mapping Between Coho Salmon (Oncorhynchus kisutch) and Three Other Salmonids Suggests a Role for Chromosomal Rearrangements in the Retention of Duplicated Regions Following a Whole Genome Duplication Event
Source: G3 (Bethesda). 2014 Jul 21;4(9):1717–30. doi: 10.1534/g3.114.012294 (PMC4169165; doi:10.1534/g3.114.012294)
Supplement: Supporting Information [file supp_g3.114.012294_FileS4.pdf]

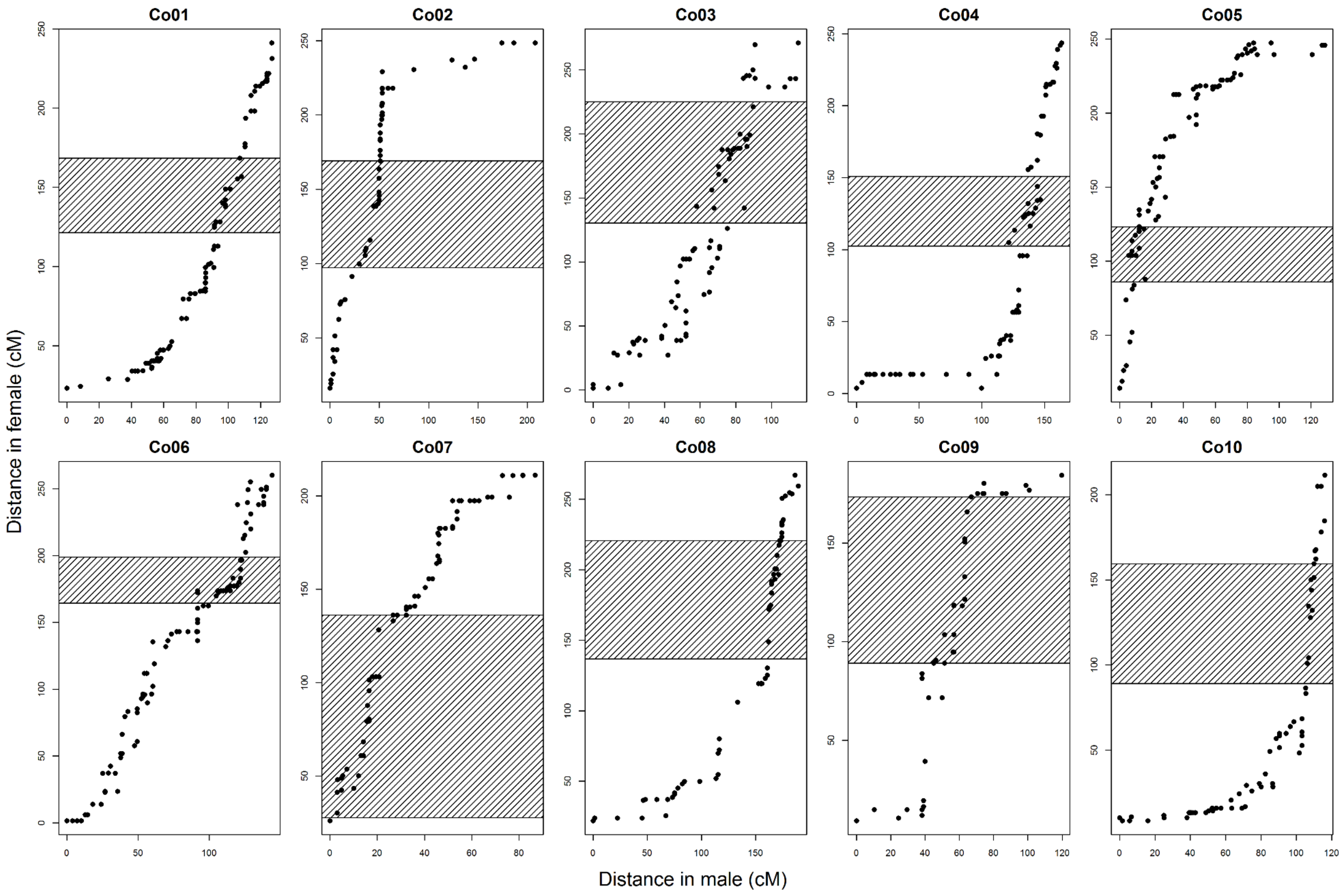
Co01
Co02
Co03
Co04
Co05
Co06
Co07
Co08
Co09
Co10
Distance in female (cM)
Distance in male (cM)

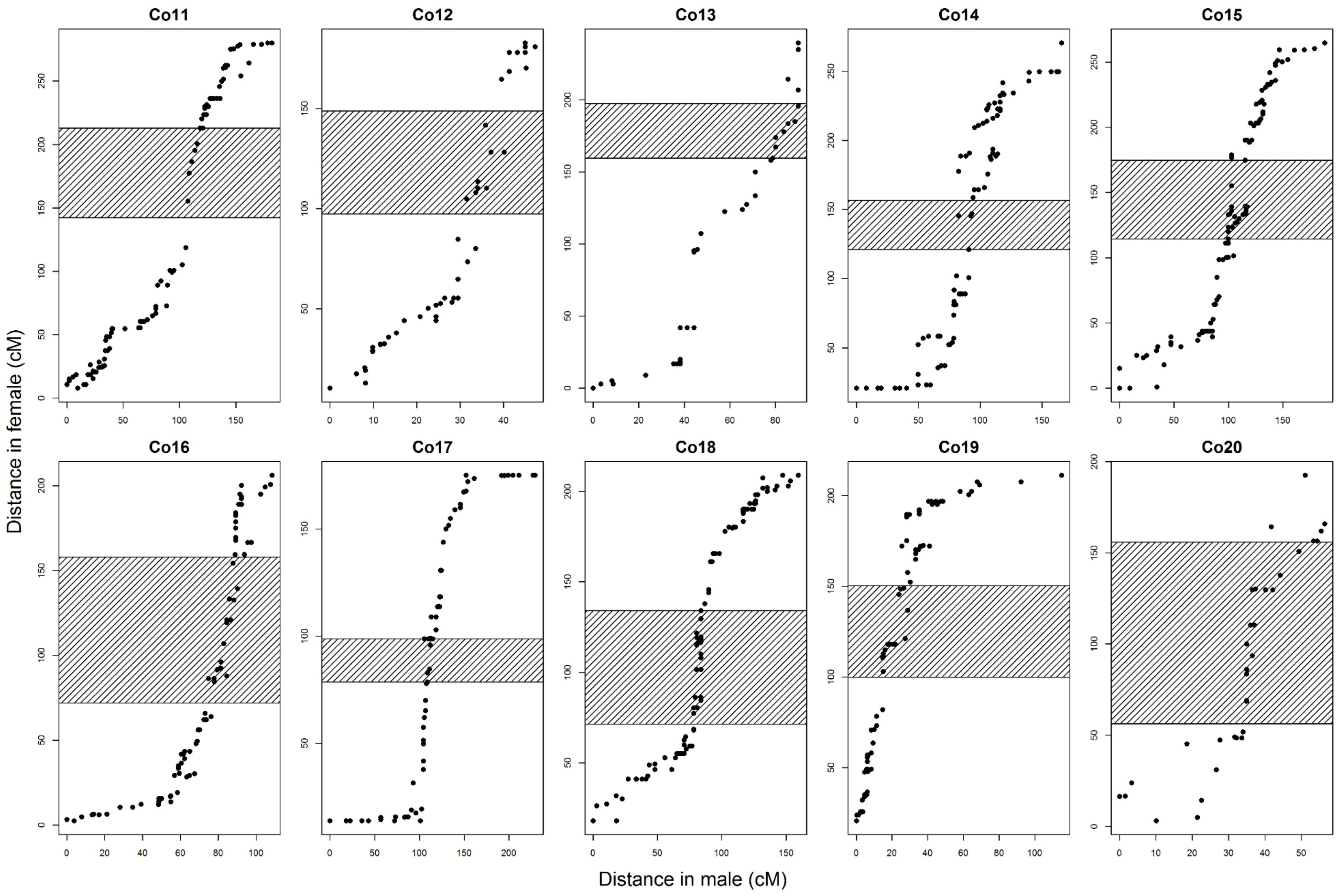
Co11
Co12
Co13
Co14
Co15
Co16
Co17
Co18
Co19
Co20
Distance in female (cM)
Distance in male (cM)

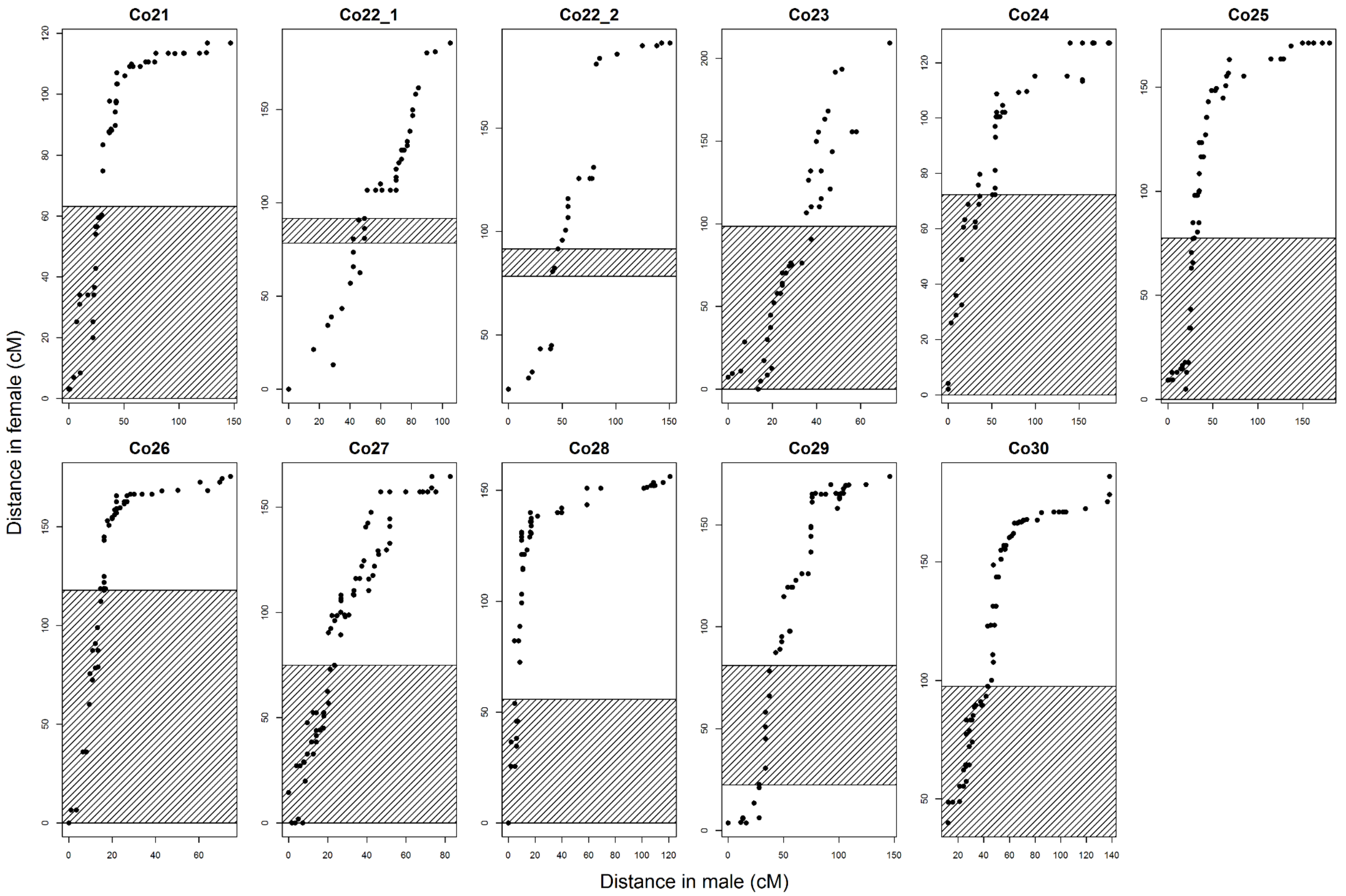
Co21
Co22_1
Co22_2
Co23
Co24
Co25
Co26
Co27
Co28
Co29
Co30
Distance in female (cM)
Distance in male (cM)

File S4: Relationship among positions of mapped RAD loci in common between the sexes. Presumed regions containing the centromere are represented by the cross-hatched area. The consensus female map constructed with haploid and diploid families (S2.2) was used for this comparison.
