# Supplementary material for: Comparative Mapping Between Coho Salmon (Oncorhynchus kisutch) and Three Other Salmonids Suggests a Role for Chromosomal Rearrangements in the Retention of Duplicated Regions Following a Whole Genome Duplication Event
Source: G3 (Bethesda). 2014 Jul 21;4(9):1717–30. doi: 10.1534/g3.114.012294 (PMC4169165; doi:10.1534/g3.114.012294)
Supplement: Supporting Information [file supp_g3.114.012294_FileS5.pdf]

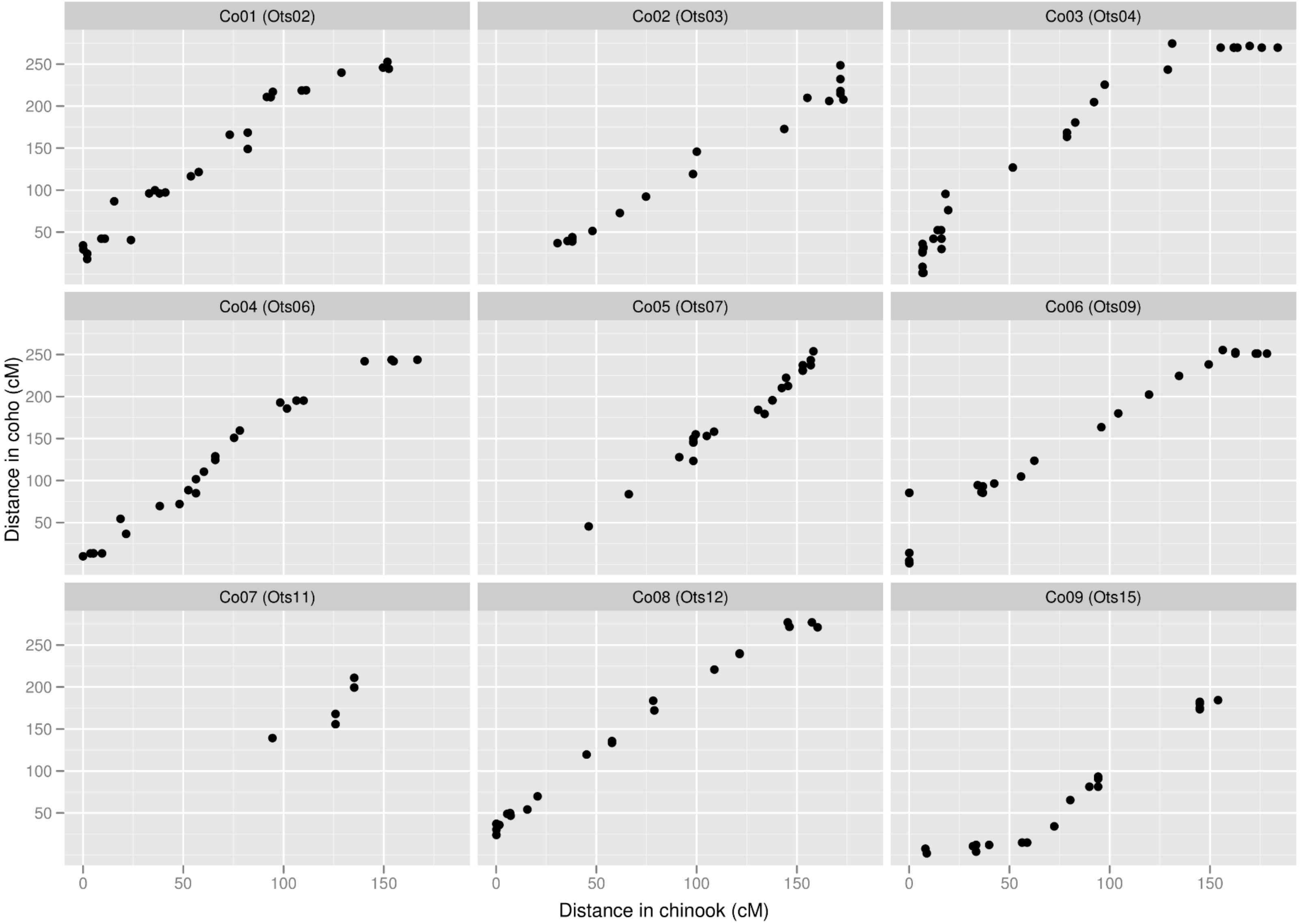

Co01 (Ots02)
Co02 (Ots03)
Co03 (Ots04)
Co04 (Ots06)
Co05 (Ots07)
Co06 (Ots09)
Co07 (Ots11)
Co08 (Ots12)
Co09 (Ots15)
Distance in coho (cM)
Distance in chinook (cM)
50
100
150
200
250
0

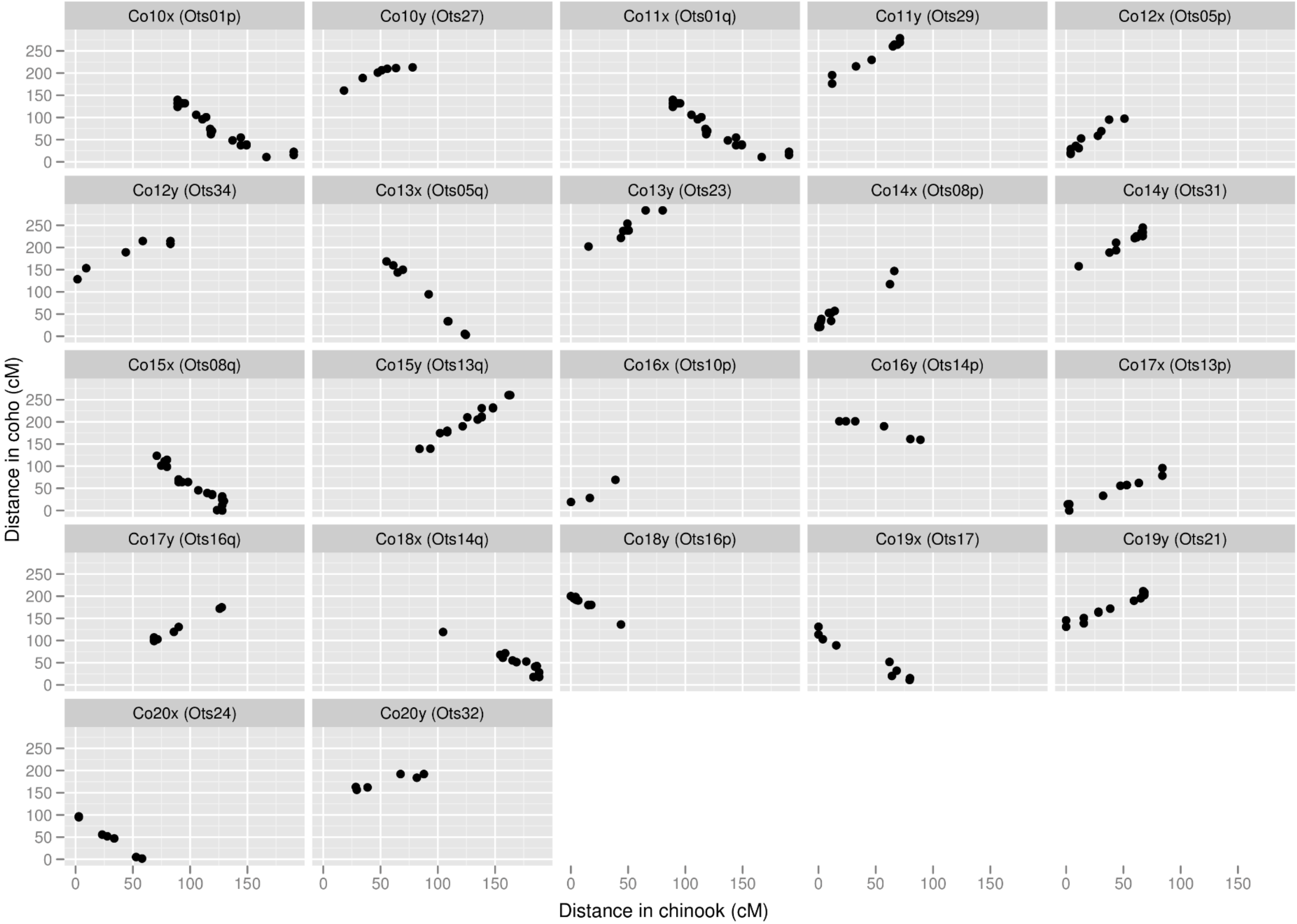
Co10x (Ots01p)
Co10y (Ots27)
Co11x (Ots01q)
Co11y (Ots29)
Co12x (Ots05p)
Co12y (Ots34)
Co13x (Ots05q)
Co13y (Ots23)
Co14x (Ots08p)
Co14y (Ots31)
Co15x (Ots08q)
Co15y (Ots13q)
Co16x (Ots10p)
Co16y (Ots14p)
Co17x (Ots13p)
Co17y (Ots16q)
Co18x (Ots14q)
Co18y (Ots16p)
Co19x (Ots17)
Co19y (Ots21)
Co20x (Ots24)
Co20y (Ots32)
Distance in coho (cM)
Distance in chinook (cM)
0
50
100
150
200
250

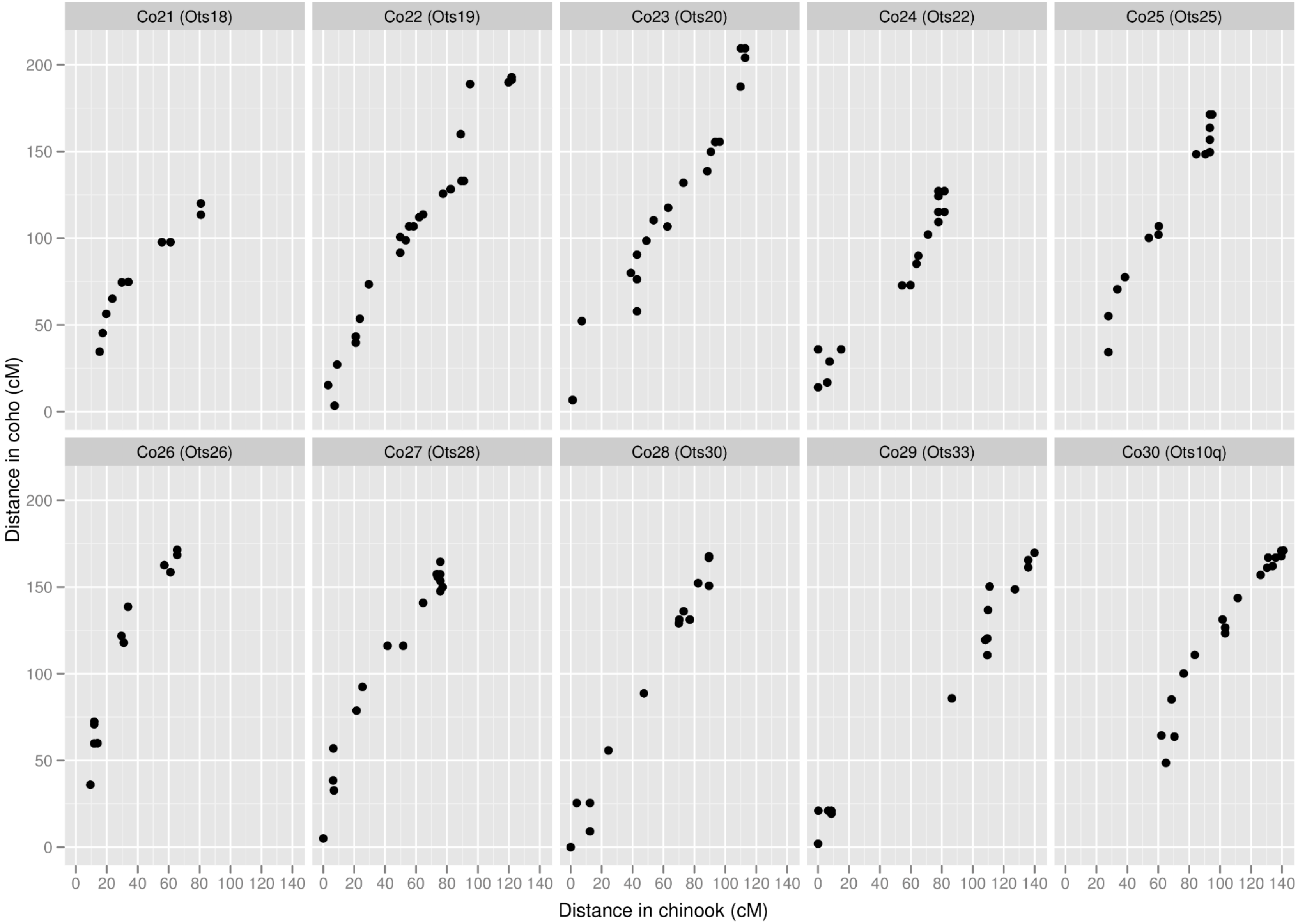

Co21 (Ots18)
Co22 (Ots19)
Co23 (Ots20)
Co24 (Ots22)
Co25 (Ots25)
Co26 (Ots26)
Co27 (Ots28)
Co28 (Ots30)
Co29 (Ots33)
Co30 (Ots10q)
Distance in coho (cM)
Distance in chinook (cM)
0
50
100
150
200
0 20 40 60 80 100 120 140

File S5: Relationship among mapped RAD loci in coho and Chinook salmon for all the linkage groups or arms.
